# Characterizing X-Ray and Solution State Conformations for a Model Qubit System: {Cr_7_Ni} Ring Rotaxanes on a Mixed Metal Triangle

**DOI:** 10.1021/acs.inorgchem.4c03919

**Published:** 2024-11-17

**Authors:** Lubomir Loci, Selena J. Lockyer, Tom S. Bennett, Ciarán J. Rogers, Adam Brookfield, Grigore A. Timco, George F. S. Whitehead, Selina Nawaz, Jack J. Miller, Richard E. P. Winpenny, Alice M. Bowen

**Affiliations:** † Department of Chemistry, The University of Manchester, Oxford Road, Manchester M13 9PL, U.K.; ‡ The National Research Facility for Electron Paramagnetic Resonance, The Photon Science Institute, The University of Manchester, Oxford Road, Manchester M13 9PL, U.K.; § The MR Research Centre and The PET Research Centre, Aarhus University, Aarhus 8200, Denmark; ∥ Department of Physics, Clarendon Laboratory, The University of Oxford, Oxford OX1 2JD, U.K.

## Abstract

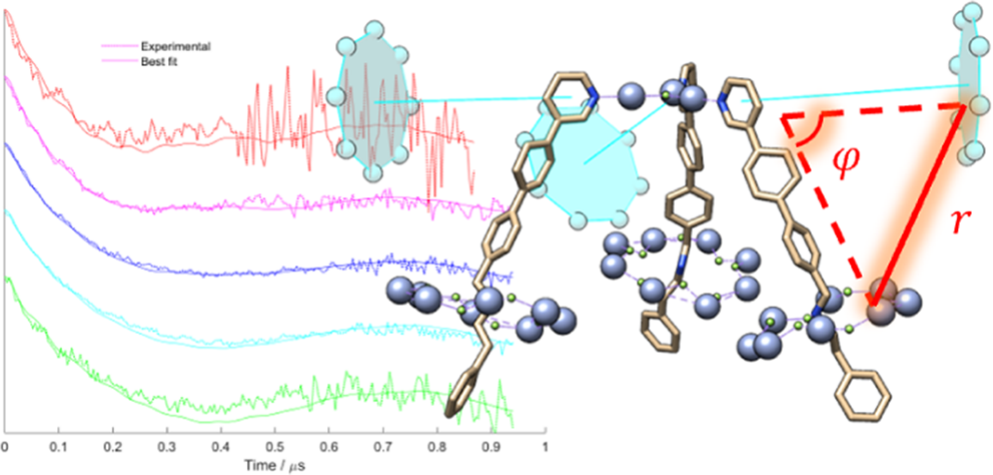

The synthesis of a series of [4]rotaxanes, each consisting of three [2]rotaxanes joined via a central {CrNi_2_} triangular linker, is reported. The resultant four [4]rotaxanes were characterized by single crystal X-ray diffraction and electron paramagnetic resonance (EPR) spectroscopy. Orientation-selective 4-pulse double electron–electron resonance (DEER) measurements between the three {Cr_7_Ni} rings incorporated in each [4]rotaxane reveal that each system is conformationally fluxional in solution, with the most abundant conformations found to differ significantly from the crystal structure geometry for each compound. The degree of similarity between conformations is evaluated using a novel application of the earth mover’s distance analysis.

## Abstract 2

A set of four macromolecular assemblies is synthesized, each containing four paramagnetic groups. Their crystal structures are determined by X-ray Diffraction and the paramagnetic groups are initially probed by continuous-wave electron paramagnetic resonance (EPR). With orientationally selective pulsed EPR, we determine the most common molecular geometries adopted by each system in solution, and the deviation thereof from the corresponding crystal structure is evaluated for each system with a novel application of the earth mover’s distance.

## Introduction

Supramolecular assemblies have become essential in a wide range of applications including MOFs,^1,2^ biomedical,^3^ catalytic,^1,4 −6^ and host–guest chemistry.^7−9^ One major advantage of supramolecular assemblies is the ability to control the size and resulting morphology, using the application of specific molecule building blocks. This makes them vital in the work toward multiqubit systems,^10,11^ and is particularly useful for the approach of multidissimilar qubit systems, where the stoichiometry and distances between each component can be tuned for a specific level of interaction.^12,13^

Many reported supramolecular assemblies result from interlocking structures, such as knots and catenanes;^14−16^ others use such rigidity for molecular capsules that enable catalytic properties.^17,18^ Rotaxanes have also been used as supramolecular building blocks; when combined with inorganic linker units, simple [2]rotaxanes can be combined to create larger, complex rotaxane assemblies.^19,20^ Characterization of such assemblies in solution is challenging when they are paramagnetic, and even more challenging where there is the potential for multiple conformations to be adopted.

One family of versatile core building blocks or nodes that have been studied in their production of large supramolecular structures are the metal triangles.^21,22^ These triangles are based on the [M_3_(μ-A)(O_2_CR)_6_] structure, where M is a transition metal that makes the core triangle shape, A is the central anion, most commonly oxide, and R is the carboxylate bridging ligand between the M atoms. The intrinsic nature of a triangle means the three metal atoms are in plane, with one site on each metal available for bonding of a monodentate ligand.

We have previously shown that using [Cr_7_NiF_8_(O_2_C^*t*^Bu)_16_]^−^ (**2**) rings we can bind a [2]rotaxane about a fluoride-centered triangle [CrNi_2_(μ_3_-F)(O_2_C^*t*^Bu)_6_(HO_2_C^*t*^Bu)_3_] (**1**) node, creating a [4]rotaxane that maintains an equilateral triangle conformation across a wide range of temperatures (3–298 K).^19^ In the previous study, the [2]rotaxanes used contained a 4-pyridine binding group on the thread, which did not allow the thread to rotate out of the plane of the triangle, leading to a relatively rigid structure where the three {Cr_7_Ni} rings lie in relatively fixed positions with their mean plane approximately perpendicular to the mean plane of the triangle.^19^ Good agreement was seen in structural data recorded by X-ray crystallography, small angle X-ray scattering (SAXS), and double electron–electron resonance (DEER), also known as pulsed electron double resonance (PELDOR),^23−25^ measured between the {Cr_7_Ni} rings, indicating a conserved structure in crystal, solution, and frozen solution states.

In this study, we use [2]rotaxane compounds that utilize a head functionalized with a nitrogen at the 3-position (relative to the thread). This leads to a far more flexible structure compared to previously studied [2]rotaxane compounds functionalized at the 4-position.^19^ Comparisons between X-ray crystallography and orientationally selective DEER (os-DEER) spectroscopy indicate that while the structure appears rigid in the crystalline state, additional flexibility is present in the solution state that is observed in frozen solution as the presence of multiple, structurally different conformers. While the presence of different conformers complicates analysis, we demonstrate that by using geometric models we can derive solution state conformations by fitting DEER traces. We additionally investigate a novel method of comparing the structural distribution in these fitted conformers: earth mover’s distance (EMD) analysis,^26^ also known as the as Wasserstein metric, Kantorovich–Rubinstein metric, or Mallows’ distance.^27^ While this has been used to quantify conformational distributions in systems such as intrinsically disordered proteins,^28^ to the best of our knowledge, it has not been employed in the sphere of conformational studies of DEER data previously.

## Results

### Synthesis and X-ray Crystal Characterization

The central node used here is the {CrNi_2_} triangle [CrNi_2_(μ_3_-F)(O_2_C^*t*^Bu)_6_(HO_2_C^*t*^Bu)_3_] (**1**), which was prepared as reported previously.^29^ To avoid repulsive steric interactions and allow binding between components, suitable threads **A**–**C** (Table 1) were prepared (see Section S1 for synthetic details). They all feature a sufficiently long spacer (>(C_6_H_4_)CH_2_) between the secondary amine site and the pyridyl (Py) headgroup in order to bind to **1**.^19^

**Table 1:**
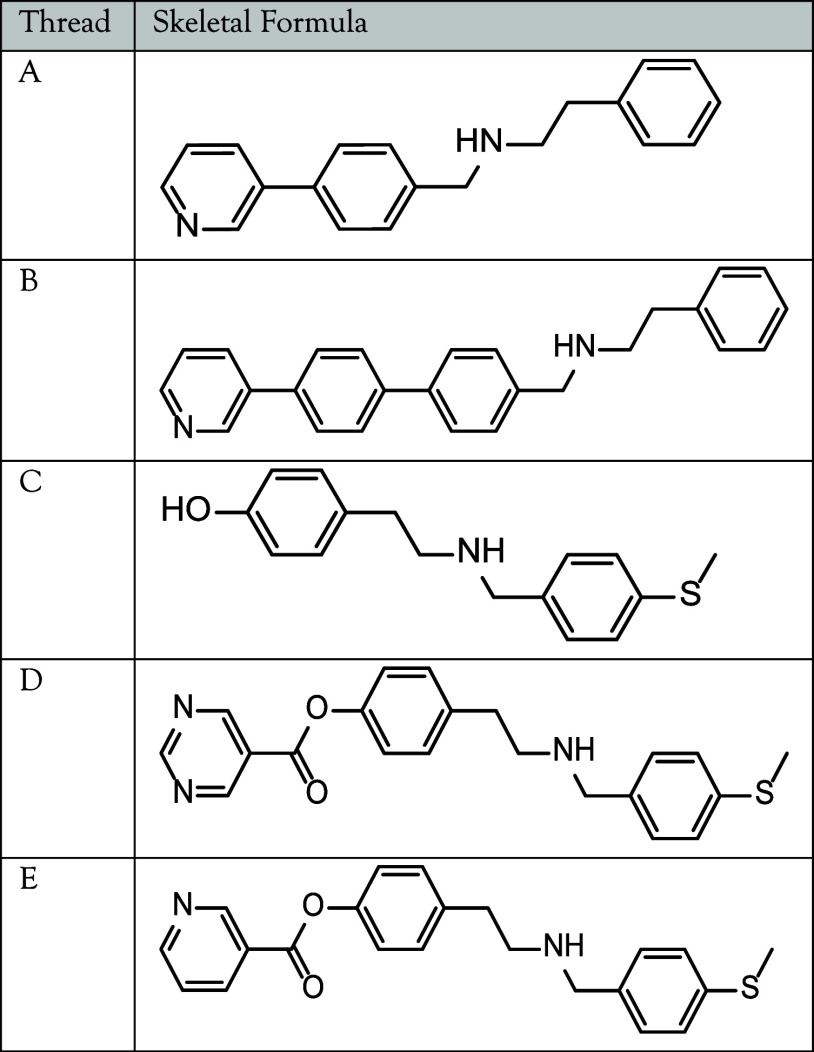
Skeletal Formula Representations of Organic Threads **A**–**E** (Generated in ChemDraw)

**Figure 1:**
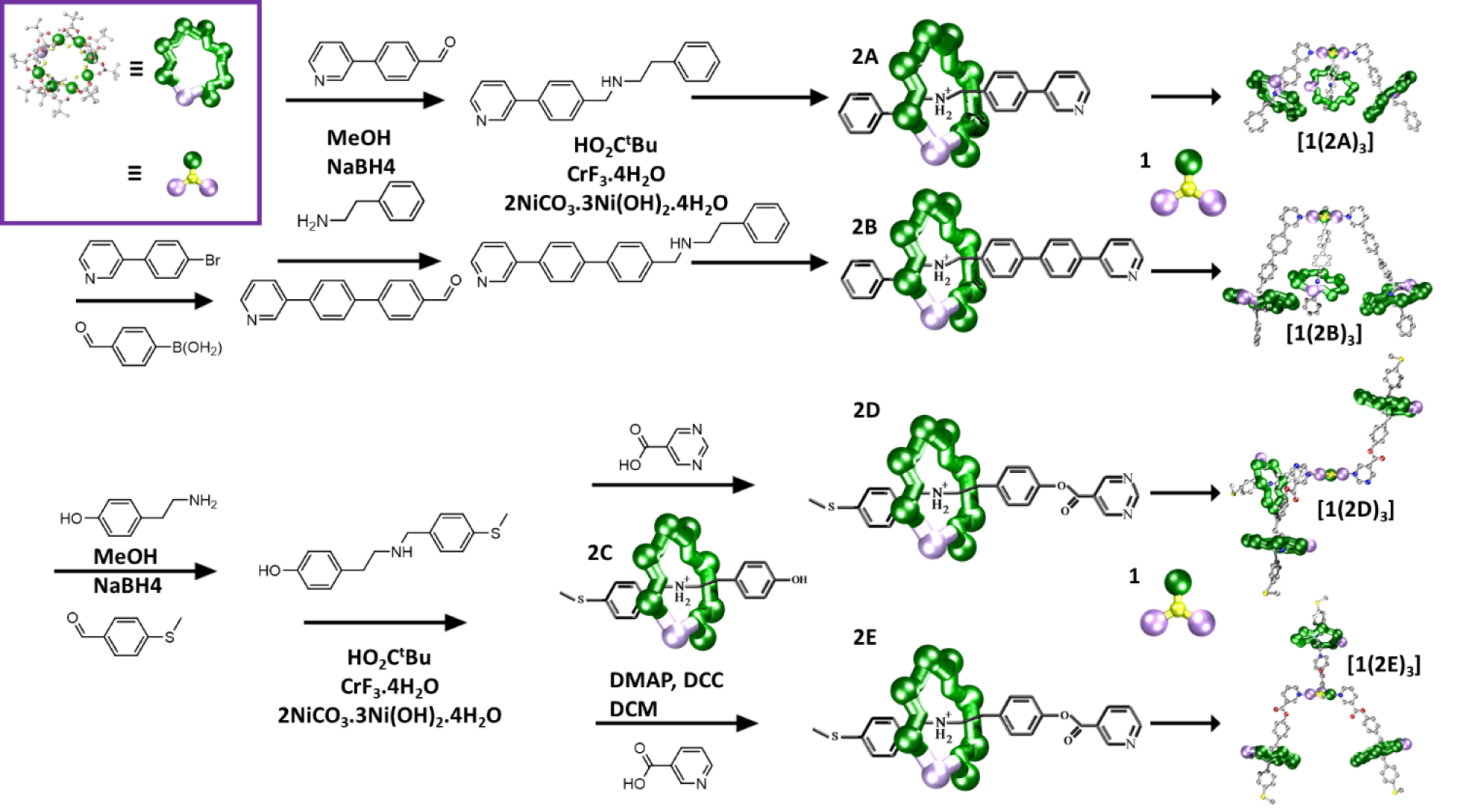
Synthetic scheme for the generation of final compounds [**1**(**2N**)_3_] where **N** = {**A**, **B**, **D**, **E**}. Atom colors: Cr, green; Ni, purple; O, red; F, yellow; N, blue; C, silver; S, dull yellow.

[2]Rotaxanes were then prepared using threads **A**–**C** respectively by reaction of hydrated chromium fluoride with basic nickel carbonate in pivalic acid in the presence of the threads (see Figure 1 and Section S1 for synthetic details). The threads are protonated at the amine site during this reaction,^30^ leading to (**A**H)[Cr_7_NiF_8_(O_2_C^*t*^Bu)_16_] (**2A**), (**B**H)[Cr_7_NiF_8_(O_2_C^*t*^Bu)_16_] (**2B**), and (**C**H)[Cr_7_NiF_8_(O_2_C^*t*^Bu)_16_] (**2C**), Figure 1.

Compound **2C** undergoes a Steglich esterification.^31^ Reaction with pyrimidine-5-carboxylic acid yields **2D** transforming **C** into **D**, and the reaction of **2C** with nicotinic acid yields **2E** that transforms **C** into **E**, resulting in (**D**H)[Cr_7_NiF_8_(O_2_C^*t*^Bu)_16_] (**2D**) and (**E**H)[Cr_7_NiF_8_(O_2_C^*t*^Bu)_16_] (**2E**), respectively, Figure 1.

The reaction of three equivalents of the [2]rotaxanes with **1** at 40 °C, in either THF (**2A**) or acetone (**2B**, **2D**, **2E**) produces the 3:1 adducts, which are [4]rotaxanes:

- [CrNi_2_(F)(O_2_C^*t*^Bu)_6_]{(**A**H)[Cr_7_NiF_8_(O_2_C^*t*^Bu)_16_]}_3_ ([**1**(**2A**)_3_]);
- [CrNi_2_(F)(O_2_C^*t*^Bu)_6_]{(**B**H)[Cr_7_NiF_8_(O_2_C^*t*^Bu)_16_]}_3_ ([**1**(**2B**)_3_]);
- [CrNi_2_(F)(O_2_C^*t*^Bu)_6_]{(**D**H)[Cr_7_NiF_8_(O_2_C^*t*^Bu)_16_]}_3_ ([**1**(**2D**)_3_]);
- [CrNi_2_(F)(O_2_C^*t*^Bu)_6_]{(**E**H)[Cr_7_NiF_8_(O_2_C^*t*^Bu)_16_]}_3_ ([**1**(**2E**)_3_]).

**Figure 2:**
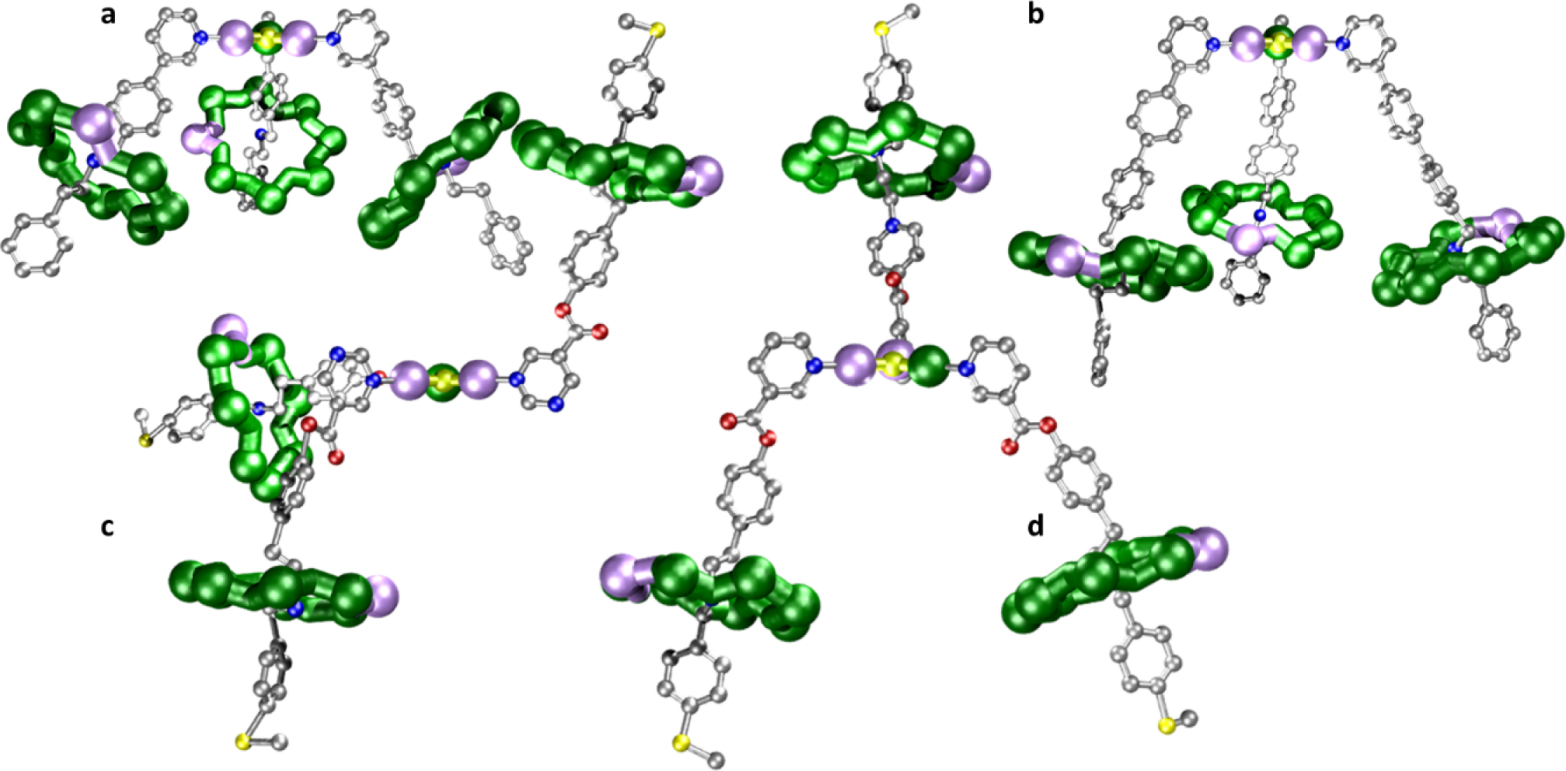
Crystal structures of [**1**(**2A**)_3_] (a), [**1**(**2B**)_3_] (b), [**1**(**2D**)_3_] (c), and [**1**(**2E**)_3_] (d). Color scheme, as per Figure 1. H atoms omitted on threads for clarity.

Crystals of the four [4]rotaxanes were all studied by X-ray diffraction (Figure 2). The structures show that while the connectivity is similar in all four [4]rotaxanes the conformation in the crystal structures varies. Metric parameters for [**1**(**2N**)_3_] where **N** = {**A**, **B**, **D**, **E**} are given in Table 2.

**Table 2:** Metric Parameters for the [4]Rotaxanes [**1**(**2N**)_3_] Where **N** = {**A**, **B**, **D**, **E**}; for **N** = **A**–**B**, There Is Only One Distance or Angle Reported Due to the C_3_ Rotation Axis Inherent in Their Crystal Structures^a^

	[**1**(**2A**)_3_]	[**1**(**2B**)_3_]	[**1**(**2D**)_3_]	[**1**(**2E**)_3_]
N^am^···F distance/Å	12.1	15.2	14.4	13.9
			13.7	14.6
			14.3	14.5
N^am^···N^am^ distance/Å	17.9	18.0	18.2	27.0
			25.1	26.2
			27.0	18.4
{Cr_7_Ni}···{CrNi_2_} angle/°	46.4	18.2	15.2	19.3
			9.4	21.7
			77.7	23.0
Py···{CrNi_2_} angle/°	89.1	87.0	88.8	84.4
			84.6	85.4
			5.6	82.7

a Angles have an error margin of ±0.3° and distances are rounded to 1 d. p.

In all compounds the [2]rotaxanes bind to the {CrNi_2_} triangle via the 3-py or pyrimidine headgroup. For the central {CrNi_2_} triangle, each metal ion is in a pseudo-octahedral geometry, with the pyridyl group of the rotaxane bound *trans* to the central μ_3_-fluoride. Each metal in the triangle is bound to four O-donors, the central μ_3_-fluoride and an N-donor from a [2]rotaxane. Each metal in the {Cr_7_Ni} ring is bound to four O-donors and two μ_2_-F, with each edge of the ring bridged by two pivalates and a fluoride. In both triangles and rings the Ni^II^ sites are disordered among the metal sites with the possible exception of in the {CrNi_2_} triangle in [**1**(**2D**)_3_].

[**1**(**2A**)_3_] crystallizes with a 3-fold axis of rotation passing through the fluoride of the triangle, therefore the asymmetric unit includes only a single [2]rotaxane, **2A** (Figure 2a). As such, the crystal structure of [**1**(**2A**)_3_] shows all 3 [2]rotaxanes veering away from the central triangle node in the same direction. In the crystal structure, the pyridyl groups are perpendicular to the {CrNi_2_} plane with an angle of 89.1° between the planes. Each {Cr_7_Ni} ring sits about the secondary ammonium group (N^am^) of (**A**H)^+^, with hydrogen bonds to two of the bridging fluorides on the interior of the ring. The plane of each {Cr_7_Ni} is made up from the core eight metal atoms and sits at an angle of 46.4° with respect to the {CrNi_2_} triangle plane.

Compound [**1**(**2B**)_3_] also crystallizes with a 3-fold rotation axis passing through the central fluoride (Figure 2b). The pyridyl groups are perpendicular to the {CrNi_2_} plane with an angle between planes of 87.0°. A difference is that each {Cr_7_Ni} plane sits at an angle of 18.2° to the {CrNi_2_} plane.

The crystal structure of [**1**(**2D**)_3_] (Figure 2c) contains the entire molecule in the asymmetric unit. Two of the pyrimidine groups bind perpendicular relative to the triangle plane, at 88.8° and 84.6°, and the third binds parallel at 5.6°. The two [2]rotaxanes where the pyrimidines are perpendicular to the plane of the triangle lead to [2]rotaxanes that point away from plane of the triangle, but in opposite directions, i.e., one “up” and one “down”. This results in the plane of these two {Cr_7_Ni} rings being close to parallel to the triangle plane with angles of 15.2° and 9.4° respectively. The third [2]rotaxane binds parallel relative to the plane of the triangle, resulting in the plane of the {Cr_7_Ni} ring sitting almost perpendicular, with an angle of 77.7°. Interestingly this structure has the possibility to yield a chiral alternative, although this has not been observed. There is a distinct difference in the N^Pyrim^···M^Tri^ distance between the two perpendicular pyrimidines and the parallel pyrimidine of 2.07 (±0.01) and 2.11 (±0.01) Å, respectively. This suggests that the Cr^III^ ion is localized in the crystal structure for the pyrimidine that binds parallel. This has been seen before in the pyridine-substituted {CrNi_2_} triangle [CrNi_2_F(O_2_C^*t*^Bu)_6_(py)_3_].^29^

The X-ray structure of [**1**(**2E**)_3_] provides yet a third conformation of the {Cr_7_Ni} rings around the {CrNi_2_} node (Figure 2d), with two of the rotaxane motifs facing down and the third facing up in the opposite direction. In the crystal structure, the angles of the pyridyl groups are marginally offset from the perpendicular to the plane of the triangle, at angles of 84.4°, 85.4°, and 82.7°. The plane of each of the {Cr_7_Ni} rings is almost parallel to that of the plane of the {CrNi_2_} triangle, with angles of 19.3°, 21.7°, and 23.0°, with the rotaxane segment that goes in the opposite direction to the other two having the smallest angle.

The crystal packing in [**1**(**2A**)_3_] shows the molecules pack almost perpendicular and alternate between rows and columns (Figure S2a). For [**1**(**2B**)_3_], each molecule stacks in the same orientation, with neighboring molecules being slightly offset. This results in the planes of all {Cr_7_Ni} and {CrNi_2_} being parallel (Figure S2b); such a crystal packing orientation could be of great interest for the application of quantum information processing (QIP) gates and algorithms where the orientation of molecules with respect to the external magnetic field is important to restrict the spread of the couplings between moieties.^32^

The crystal packing of [**1**(**2D**)_3_] (Figure S2c) shows the molecules aligning in the same direction, but with each alternating row rotated by 180°, with the localized Cr^III^ ion in the {CrNi_2_} triangle swapping from the left to right, relative to the central fluoride. The crystal packing of [**1**(**2E**)_3_] (Figure S2d) is similar to [**1**(**2D**)_3_], but in [**1**(**2E**)_3_], the rows alternate from two rings pointing above the {CrNi_2_} plane and one pointing below it, to two rings pointing below the {CrNi_2_} plane and one pointing above it. Like [**1**(**2B**)_3_], this results in all the planes (and *g*_*z*_ components) of the {Cr_7_Ni} rings and {CrNi_2_} being almost completely parallel to one another; however, the difference in inter-ring distances in each molecule provides additional spread in the dipolar couplings.

We have previously used small-angle X-ray scattering (SAXS) combined with molecular dynamics simulations (MDS) in parallel with DEER.^19^ Here we find MDS do not converge and all SAXS data recorded appears similar, only confirming the size of the molecule in solution with no directly interpretable information on internal structure (see Section S4 for further details). As both MDS and SAXS are uninformative, we have extended the pulsed EPR analysis.

### Continuous Wave (cw) EPR Spectroscopy

**Figure 3:**
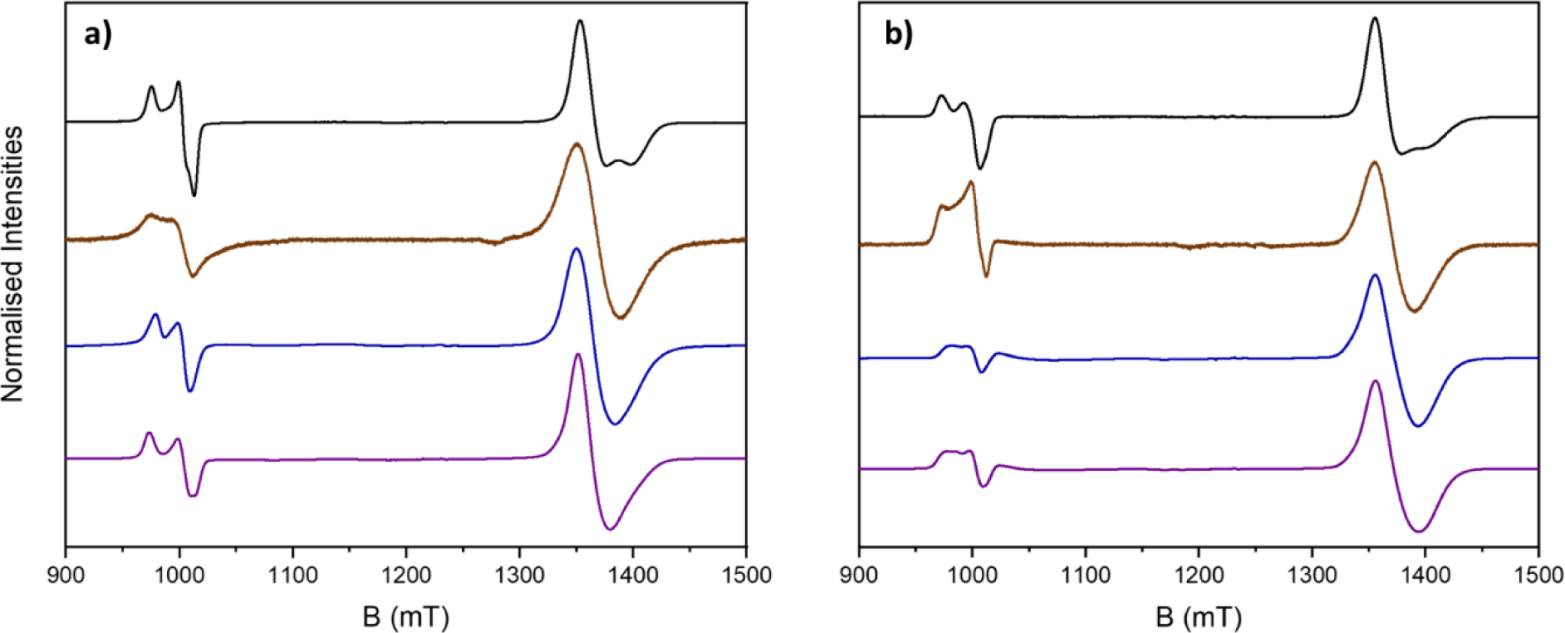
CW (Q-band, ca. 34 GHz) experimental EPR spectra of [**1**(**2A**)_3_] (black), [**1**(**2B**)_3_] (brown), [**1**(**2D**)_3_] (blue), and [**1**(**2E**)_3_] (purple) as a powder (a) and solution (b) at 5 K.

Continuous-wave (cw) Q-band (ca. 34 GHz) electron paramagnetic resonance (EPR) spectroscopy measurements were performed on [**1**(**2N**)_3_] (**N** = {**A**, **B**, **D**, **E**}) at 5 K for powder samples and for frozen 3 mM 1:1 DCM/toluene solutions. At 5 K resonances are seen at *g* ≈ 1.78 and *g* ≈ 2.48. From precedent, these can be assigned to the *S* = 1/2 ground states of the {Cr_7_Ni} and {CrNi_2_} units respectively (Figure 3). No interaction between the *S* = 1/2 states can be seen by cw EPR spectroscopy. The spectra were simulated using a simple spin Hamiltonian incorporating only the individual *g*-matrices for the {Cr_7_Ni} signal and the {CrNi_2_} signal, with *g*-strain applied where appropriate:
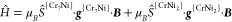


The *g*-values are given in Table 3, as well as in the Tables S1 and S2, and simulations are shown in Figures S5 and S6.

**Table 3:** Parameters Used to Simulate cw EPR Spectra^a^

	Powder		Solution	
	*g*_*xyz*_ {Cr_7_Ni}	*g*_*xyz*_ {CrNi_2_}	*g*_*xyz*_ {Cr_7_Ni}	*g*_*xyz*_ {CrNi_2_}
[**1**(**2A**)_3_]	1.785(24)	2.422	1.785(15)	2.425
	1.785(24)	2.395	1.785(15)	2.415
	1.735(24)	2.492	1.730(30)	2.498
[**1**(**2B**)_3_]	1.785(30)	2.420	1.785(25)	2.410(20)
	1.785(30)	2.400	1.785(25)	2.405(20)
	1.745(30)	2.490	1.730(25)	2.495(20)
[**1**(**2D**)_3_]	1.795(30)	2.423	1.787(30)	2.420(25)
	1.795(30)	2.415	1.787(30)	2.415(25)
	1.750(30)	2.490	1.738(30)	2.485(30)
[**1**(**2E**)_3_]	1.790(25)	2.423	1.782(27)	2.412(20)
	1.790(25)	2.400	1.782(27)	2.412(20)
	1.750(25)	2.495	1.737(27)	2.490(25)

a Where *g*-strain was used the value is given in parentheses. All spectra had a Lorentzian line width of 10 mT except the powder spectrum of [**1(2A)**_**3**_], which had a Gaussian line width of 5 mT.

The resolution of the spectra differ between the four [4]rotaxanes, and in some cases between solution and powder. The spectra of [**1**(**2A**)_3_] are noticeably narrower than those of the other three, and as a result in both solution and powder spectra of [**1**(**2A**)_3_] the *g*_*z*_ component of the *S* = 1/2 resonance due to the {Cr_7_Ni} ring is resolved. The splitting of the {CrNi_2_} feature (*g*_*z*_ > *g*_*x*_ ≈ *g*_*y*_) is also more pronounced for [**1**(**2A**)_3_].^29^

The difference between the powder and solution spectra is more pronounced for [**1**(**2N**)_3_] (**N** = {**B**, **D**, **E**}), and is mainly seen in the broadening of the splitting for the *g*_*z*_ and *g*_*x*_/*g*_*y*_ of the {CrNi_2_} signal. [**1**(**2D**)_3_] shows the highest degree of broadening in solution; this is confirmed by spectral simulation (see Figures S5 and S6, Tables S1 and S2), with the largest amount of *g*-strain required to optimally simulate the solution spectra.

The spectra of [**1**(**2N**)_3_] (**N** = {**D**, **E**}) show a less intense *S* = 1/2 ground state signal for the {CrNi_2_} triangle (relative to the {Cr_7_Ni} ring) than those of [**1**(**2N**)_3_] (**N** = {**A**, **B**}). This is due to the presence of a low-lying *S* = 3/2 excited state, which is usually seen more at higher temperatures or higher microwave frequencies.^29^ The low-intensity *S* = 3/2 transition is centered around 1160 mT (*g* = 2.1) and features in both the solution and powder spectra for [**1**(**2N**)_3_] (**N** = {**D**, **E**}). It is present for [**1**(**2N**)_3_] (**N** = {**A**, **B**}) but is much less pronounced and barely visible, and as such does not impact the intensity of the *S* = 1/2 signal.

### Pulsed EPR Spectroscopy

**Figure 4:**
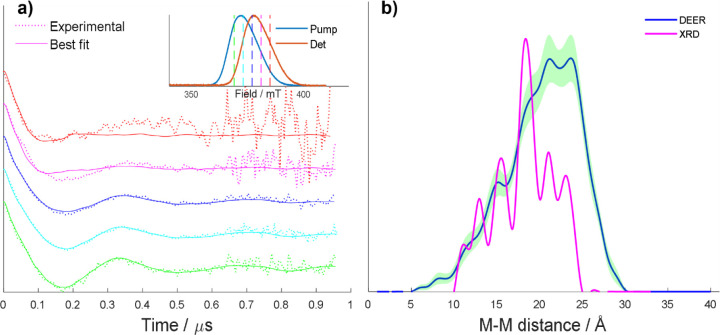
(a) Normalized experimental DEER time traces (dashed lines) of [**1**(**2A**)_3_] overlaid with the simulated conformation fits (solid lines), see Section S7 for details of the fitting procedure. The experimental traces are generated by stitching together two traces of different length to provide better signal-to-noise close to the zero time. This leads to a change in noise level in the last part of the trace, above 0.65 μs. Inset: experimental field sweep spectra obtained for [**1**(**2A**)_3_] at the pump and detection frequencies, with each dashed vertical line indicating the field position of the time trace with the same color. (b) Inter-ring metal–metal distance distributions of [**1**(**2A**)_3_] (smoothed using cubic-spline interpolation in MATLAB) in solution (blue, sum of best-fitting 50 conformations) and in its crystal structure (magenta, normalized to the maximum of the solution-phase distribution sum); the 95% confidence range for the solution-phase distribution is highlighted in green.

To determine whether the crystalline molecular geometries of compounds [**1**(**2N**)_3_] (**N** = {**A**, **B**, **D**, **E**}) are retained in solution, orientation-selective double electron–electron resonance (DEER) – a pulsed EPR technique that probes the coupling interaction between unpaired electrons – has been conducted on frozen solutions of each of these compounds at 3 K. An offset frequency of +150 MHz between the probe pulses and pump pulse was used in all cases, and the experiment repeated at several field positions (Figure 4a inset) to probe the complete {Cr_7_Ni} ring spectrum, corresponding to different orientations of the rings and associated dipolar coupling vectors with respect to the external magnetic field (see Section S6 for additional experimental details). In a DEER experiment, the spin echo amplitude is monitored as a function of the position in time of the pump pulse. The modulation of the echo by the dipolar coupling manifests itself as oscillations in the time trace, as can be observed for the traces of compound [**1**(**2A**)_3_] (Figure 4a). For the systems studied here, due to the large – ca. 20–30 Å – through-bond inter-ring distances present, as well as the lack of an extended π-conjugation network connecting the rings, the {Cr_7_Ni}···{Cr_7_Ni} coupling interaction is assumed to be a purely dipolar interaction, with the exchange contribution to the coupling taken to be negligible. Such an approximation has been shown to be acceptable for similar systems.^19,33^

Dipolar interactions between electron spins depend on both distance and relative orientation; since the pump and detection pulses excite {Cr_7_Ni} rings in different regions of the field sweep spectra (see inset in Figure 4a for an example), the resultant data therefore contains information on both the inter-ring distance and the relative orientation of the rings with respect to each other.

While in cases where there is little to no orientation selection and little to no correlation between the orientation of the *g*-tensor and that of the dipolar spin–spin vector, reliable interspin distance distributions can be extracted from the oscillations using a Tikhonov regularization,^34^ this is not possible for individual traces recorded on these systems. However, provided multiple traces at various magnetic fields – sampling a sufficient range of orientations – can be acquired, analysis of the data can determine the distance and orientation of the spin–spin vector relative to the *g***-**tensors of the unpaired electron spins, by fitting the data to geometric models.^35−37^

A set of five DEER traces was measured for each of the four compounds, with each trace measured at a different field value, corresponding to excitation of different ring orientations with respect to the magnetic field (see Section S8 for DEER traces of compounds [**1**(**2N**)_3_] (**N** = {**B**, **D**, **E**})). As it is more important to fit the early section of the time traces correctly (where the oscillations are more pronounced), to improve the signal-to-noise of this early region, a shorter time trace and a longer time trace was recorded for compounds [**1**(**2N**)_3_] (**N** = {**A**, **B**, **E**}); the shorter and longer traces were subsequently stitched together using the DEERStitch algorithm.^38^ This also follows the ideas of the relaxation-optimized acquisition length distribution (RELOAD) scheme.^39^

DEER traces generated from the crystal structures of compounds [**1**(**2N**)_3_] (**N** = {**A**, **B**, **D**, **E**}) differ considerably from the experimental traces (Section S7 and Figure S9), indicating deviation from the crystal structure when in solution. In order to determine the most abundant conformations adopted by the compounds in solution, 3000–5000 potential conformers were initially generated for each compound based on a geometric model of molecular flexibility about the N^Py^···M^Tri^ bond, based on conical rotation, with the ring *g*-tensor orientations assigned based on previous studies^33,40^ (see Section S7 for precise details of the model employed); a set of DEER traces was then simulated for each of the generated conformers, using the parameters used experimentally, to create a library of simulated DEER traces. The acquired experimental traces were fitted to the library of simulated traces (Figure 4a) using least-squares fitting and an iterative fitting procedure (see Section S7 for details). In each case, no significant improvement in the quality of the fit was observed beyond 50 fitted conformations.

All inter-ring metal–metal distances were recorded for each conformation included in each best fit to yield histograms and these were compared with analogous distributions obtained from the crystal structure of each compound, as illustrated for compound [**1**(**2A**)_3_] (Figure 4b). This was carried out to qualitatively investigate the degree of similarity between these distances in the crystal structure and in the solution phase for each of the four compounds.

**Figure 5:**
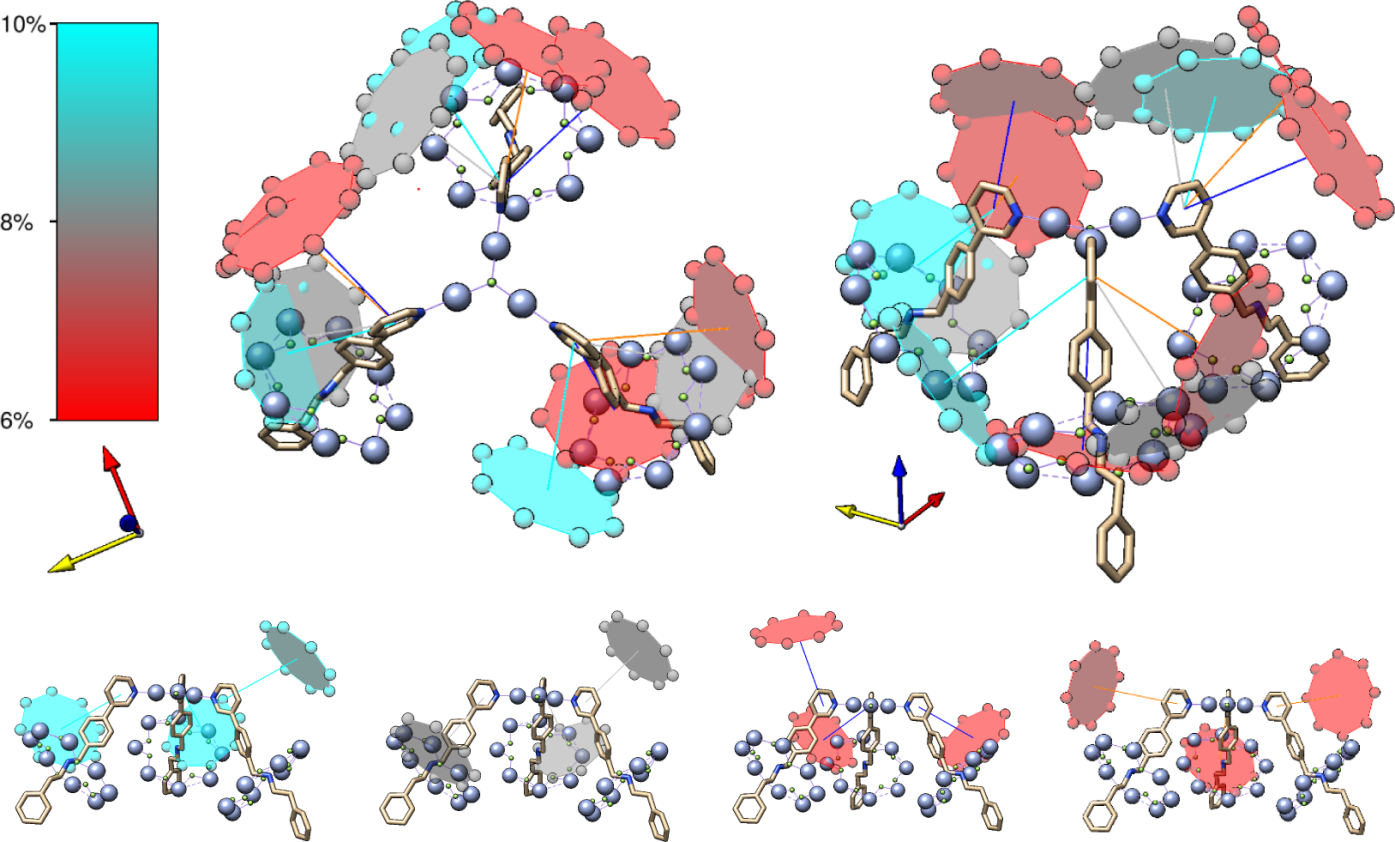
Crystal structure of [**1**(**2A**)_3_] overlaid with the most dominant (≥6%) solution-phase conformations (shown from two different points of view, as well as separate individual conformations below), with the ring color reflecting the abundance of the conformation in the fit (see color key on the left) and the organic threads of each conformation depicted by lines of the same color; the g-frame of the central {CrNi_2_} triangle is shown by the axes alongside each structure (*x*: red, *y*: yellow, *z*: blue).

For compound [**1**(**2A**)_3_], the frozen solution-phase distance distribution has components at notably larger distances than the crystalline phase distance distribution, suggesting that there is a drive for the rings to move away from each other in solution via rotation of the organic threads about the N^Py^···M^Tri^ bond. In addition, the maxima of the frozen solution-phase distribution are less pronounced compared to the crystallographic distribution, indicative of the wider range of conformational geometries adopted by the compound in solution. A similar – and even more pronounced – result is found for compound [**1**(**2B**)_3_]. By comparison, the width of the frozen solution-phase distributions of compounds [**1**(**2D**)_3_] and [**1**(**2E**)_3_] appear to be more similar to those obtained from their crystal structures (see Figures S10, S12, and S14 for distance distributions of compounds [**1**(**2N**)_3_] (**N** = {**B**, **D**, **E**})), although again the solution-phase maxima are less pronounced. This can be explained by their crystal structures: both [**1**(**2A**)_3_] and [**1**(**2B**)_3_] adopt a crystal-phase geometry with a 3-fold rotation axis where all three [2]rotaxanes veer away from the central {CrNi_2_} triangle in the same direction (see Synthesis and X-ray Crystal Characterization section). This brings the {Cr_7_Ni} rings close to each other in the crystal phase, creating a drive for the rings to move away from each other – due to steric as well as electrostatic repulsions – in the solution phase where the conformational flexibility is much greater. [**1**(**2D**)_3_] and [**1**(**2E**)_3_], on the other hand, adopt crystal structures where the rings are more distant from each other, reducing the incentive for further ring separation. As well as comparing the inter-ring metal–metal distances, the most dominant conformations of each compound in the solution phase can be visualized and compared to its crystal structure to obtain a qualitative picture of how the conformational geometries differ from each other in space. In solution, [**1**(**2A**)_3_] (Figure 5) mostly tends to adopt conformations where one of the [2]rotaxanes veers away from the {CrNi_2_} triangle in the opposite direction to the other two (i.e., in a “two-up-one-down” arrangement), while [**1**(**2B**)_3_] (Figure S11) displays a strongly dominant conformation, where all three {Cr_7_Ni} rings are close to or in line with the plane of the triangle. Although [**1**(**2D**)_3_] and [**1**(**2E**)_3_] both adopt a range of conformations in solution (Figures S13 and S15), those of [**1**(**2E**)_3_] appear to be more similar to the crystal structure than is the case for [**1**(**2D**)_3_].

## Discussion and Analysis

Results from modeling the DEER data sets indicate that the most common conformations in solution do not necessarily possess similar geometries to the ones adopted by each compound in the crystal phase (see Section S7 and Section S8), and much greater structural variation is seen in the frozen solution phase compared to the crystalline phase. This study thus supports the view that the conformational geometries adopted by a compound in solution or frozen solution should not be assumed to resemble the crystal packing geometries observed via X-ray diffraction studies without any further studies conducted in these phases.

To investigate these structural differences quantitatively, it becomes necessary to find a method, by which the geometric similarity of two conformations (or groups of conformations) with respect to each other can be determined. A novel application of the earth mover’s distance (EMD) was employed for this purpose, using methodology previously developed for image retrieval.^41,42^ The EMD is a statistical parameter quantifying the minimum movement or work required to transform one distribution into another (see Section S9 for more details on the EMD). By calculating the EMD between the positions of the rings, when the position of the triangle is aligned and centered at (0,0,0), in the crystal structure of a system and in its best-fitting solution-phase conformations (determined from fitting the DEER traces), the geometric similarity of the crystal structure to the solution state conformations can be described. EMD analysis can therefore be used to carry out a structural comparison of each system in the crystal phase and in the solution phase.

All EMD values in this paper have used the coordinates of the ring centers for each system as the basis set for analysis and comparing these with respect to each other. For each conformer of the best fit, six orientations of the conformer, related by rotation about a *C*_3_ axis perpendicular to the plane of the triangle and three *C*_2_ axes in the plane of the triangle bisecting through one metal center, were considered. In each case, a ground distance between each orientation of the conformer and the crystal structure was calculated, and the lowest value used to contribute to the calculated EMD. Based on its definition, the EMD calculated here is the Wasserstein metric; the lower the EMD value, the greater the similarity between the two conformation sets being compared (with an EMD of 0 indicating the two sets are identical). In this comparison, the weight of the XRD structure is set to 50 times the weight of a single contribution to the best fit library, which is made up of 50 structures, making the mass of each distribution equal such that the EMD is a true metric, considering both distribution and probability.

It is important to note when comparing EMD values between different compounds that the EMD is affected by the thread length, since this influences the inter-ring distances observed. To allow for this when comparing EMD values between systems, each EMD value has been multiplied by a scaling factor equal to the quotient of the mean triangle-ring distance for compound [**1**(**2A**)_3_] and the analogous mean distance for the compound in question, scaling the EMD values of the other three compounds with respect to compound [**1**(**2A**)_3_]. This is a reasonable scaling factor to take as the distances we are interested in are essentially chords (*C*) on a circle related to the radius of the circle (the triangle-ring distance, *R*) by *C* = 2*R* sin(θ/2), where θ is the angle made between the two triangle-ring vectors. As we have used the same angular dependence to make each geometric model, the possible ranges of θ are constant for each system and hence *C* ∝ *R*. The mean triangle-ring distances and scaling factors used are provided in Section S9 (Table S7).

The results presented in Table 4 show that the conformational geometry of compound [**1**(**2B**)_3_] in solution appears to deviate the most from its crystal structure geometry, while that of compound [**1**(**2E**)_3_] shows the least deviation. In all cases, there is significant deviation between the structures adopted in each of the two phases.

**Table 4:** Scaled Mean EMD Values (Rounded to 4 d. p.) between the Crystal Structure and the 50 Best-Fitting Simulated Solution-Phase Conformations for Each Compound

Compound	XRD vs Best-fit ⟨EMD⟩ (scaled)
[**1**(**2A**)_3_]	0.7377
[**1**(**2B**)_3_]	0.9820
[**1**(**2D**)_3_]	0.8288
[**1**(**2E**)_3_]	0.6317

It is also possible to calculate the EMD considering the spherical polar angles (φ and θ) made between the ring-triangle vector and the vector described by the corresponding N^Py^···M^Tri^ bond. Mean EMD values have been calculated individually for both θ and φ for each compound (see Table 5), with θ being the angle between the ring-triangle vector and the N^Py^···M^Tri^ bond vector (taken as the new *z*-axis) and φ being the counterclockwise angle between the projection of the ring-triangle vector onto the new *xy*-plane and the new *x*-axis. This new method of calculation has the advantage of more accurately describing how any two conformations would in fact interconvert (via rotation about the N^Py^···M^Tri^ bond), rather than calculating the conformational difference purely from the distances between the rings of each conformation and ignoring any angular information encoded in the conformers. In the case of considering the angles it is not necessary to scale the EMD values between conformers in the same way as when distance information is considered.

**Table 5:** Mean Angular EMD Values (Rounded to 4 d. p.) for Angles φ and θ between the Crystal Structure and the 50 Best-Fitting Simulated Solution-Phase Conformations for Each Compound

	XRD vs best-fit angular ⟨EMD⟩	
Compound	φ	θ
[**1**(**2A**)_3_]	1.2723	0.1539
[**1**(**2B**)_3_]	1.2752	0.2307
[**1**(**2D**)_3_]	1.0101	0.2399
[**1**(**2E**)_3_]	0.7702	0.1864

In each case, the new *x*-axis was chosen to lie parallel to the projection of the crystal structure ring-triangle vector onto the new *xy*-plane, i.e., so that the φ angle of each crystal structure ring-triangle vector is set to 0. This ensures that the EMD calculated between the crystal structure and any single simulated conformation is a valid measure of angular difference between the two geometries and does not give erroneous values stemming from the convention, by which the sign of φ is defined based on which spatial region the corresponding ring-triangle vector is located in (see Section S10 for further explanation).

In spherical polar coordinates, φ defines the rotation of the ring-triangle vector about the N^Py^···M^Tri^ bond vector; as such, the EMD values for the φ angles accurately describe similarity of conformations. On the other hand, θ defines the elevation angle between the ring-triangle vector and the N^Py^···M^Tri^ bond vector. Since the allowed range of values for this angle was manually set by our geometric flexibility model (Section S7 and Figure S8), the EMD values for θ only describe the variation in this angle within the limits set by the model, and based on the definitions of the two angles, it is the EMD values for φ that give an insight into the rotational flexibility of the systems studied. Thus, investigation of the angular EMD in this study is mostly restricted to discussion of the EMD values for φ (rather than θ) and their implications for the conformational flexibility of each system.

In agreement with the distance-based EMD measurements (Table 4), compound [**1**(**2B**)_3_] in solution deviates the most from its crystal structure, while compound [**1**(**2E**)_3_] deviates the least. However, the angular EMD values for compounds [**1**(**2A**)_3_] and [**1**(**2D**)_3_] are not in line with the corresponding distance-based EMD values. Since our geometric model of flexibility assumes the [2]rotaxane components of the molecule can all freely rotate about the N^Py^···M^Tri^ bond, the angular EMD values – calculated based on rotation angles – are likely to be the more valid and accurate form of similarity measurement.

**Figure 6:**
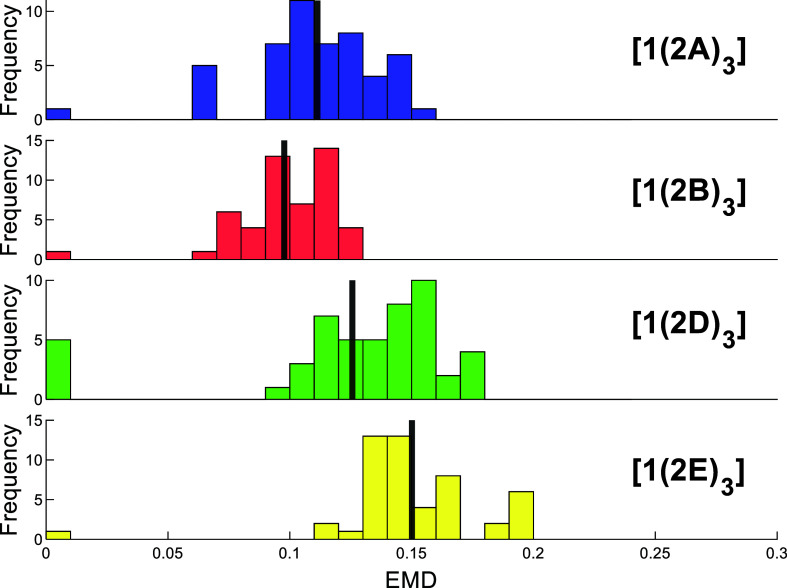
Distributions of scaled EMD values between the original fit (50 best-fitting conformations) and each of the forced fits (obtained on making the fit start with a specific conformation in the original fit) for [**1**(**2N**)_3_] (**N** = {**A**, **B**, **D**, **E**}); the mean EMD value is indicated by the vertical black line for each compound.

The EMD analysis can also be used to investigate the robustness of the fitting procedure, as it is known that the iterative fitting method is sensitive to the first geometry chosen as a fit. Comparison of the original best-fit conformations to the best-fit conformations obtained when the fitting is made to start with a specific conformer in the original fit (termed “forced fit”) was performed. For each conformer *n* in the original fit, a forced fit was obtained by starting the fitting with conformer *n*. Subsequently, the 50 forced fits generated were compared to the original fit and their likeness was evaluated by counting the number of conformations that appeared in both fits, as well as by calculating the mean distance-based EMD between the best fit and each of the forced fits (see Table 6 and Figure 6). Similarly to the EMD measurements between the crystal structures and the solution-phase conformations (Table 4), the mean EMD values between the best fit and the forced fits were scaled to remove the dependence of the EMD on thread length. In each case, there are 50 conformers considered, making the weight of each complete distribution the same and the EMD a true metric. The average EMD value for each compound in this case describes the robustness or validity of the fit, with a lower EMD indicating a more robust fit as lower EMD values correspond to greater similarity between the original fit and the forced fits generated on starting the fit with a specific conformer. As discussed earlier with the EMD calculations between the crystal structure and the best-fitting conformations in solution, angular EMD calculations may give incorrect results; this arises from the convention in which φ is defined as being positive or negative in different spatial regions. Hence, these EMD calculations were not performed with the angular parameters as we do not have confidence that the angular EMD values obtained would not deviate significantly from their true values due to this source of error.

**Table 6:** Average Number of Conformations Present in Both the Original Fit and the Forced Fits, and the Scaled EMD Value between the Original Fit and the Forced Fit – Averaged across 50 Forced Fits – for Each Compound

Compound	Average retained conformers	⟨EMD⟩ (scaled)
[**1**(**2A**)_3_]	34.5	0.1113
[**1**(**2B**)_3_]	39.78	0.0977
[**1**(**2D**)_3_]	32.48	0.1256
[**1**(**2E**)_3_]	25.64	0.1501

With this analysis method, a clear trend in similarity is observed across the four compounds ([**1**(**2B**)_3_] > [**1**(**2A**)_3_] > [**1**(**2D**)_3_] > [**1**(**2E**)_3_]), with the fit for [**1**(**2B**)_3_] having the highest validity and the fit for [**1**(**2E**)_3_] being the least robust. The same trend is echoed if we look at the average number of conformers retained in each fit, which is greatest for [**1**(**2B**)_3_] and lowest for [**1**(**2E**)_3_]. The width of the distribution of EMD values found from this validation methodology is also lowest for compound [**1**(**2B**)_3_] (Figure 6). Taken together, these results provide additional validation that the solution-state structure for compound [**1**(**2B**)_3_] is considerably different from that found in the crystal structure.

## Conclusions

We have prepared a set of [4]rotaxanes by connecting three [2]rotaxanes with a central triangular linker via organic threads functionalized on one end by a pyridyl or pyrimidyl headgroup in each case. The four resulting paramagnetic supramolecular assemblies (labeled [**1**(**2N**)_3_] where **N** = {**A**, **B**, **D**, **E**}) have been confirmed to adopt different crystal structures and/or packing arrangements via X-ray diffraction, illustrating the key role played by the identity of the organic thread in determining the behavior of these compounds in their crystalline phases.

Pulsed EPR studies conducted on frozen solutions of these compounds indicate that all four systems exhibit a degree of conformational fluxionality in solution, present in the frozen solution as an array of conformations, and that the crystal structures adopted by the compounds are not necessarily representative of the dominant conformations they adopt in solution. The results of this study have implications for the potential application of these systems as molecular qubits: since a range of conformations was found to be present in the solution phase for each compound, the molecules are evidently conformationally fluxional in solution. This would hinder the potential to selectively address the rings, since the resonance frequency of the {Cr_7_Ni} ring depends on the relative orientation of the ring with respect to the applied magnetic field, and the fluxional behavior would lead to constant fluctuations in the signal frequency required to address a particular ring. It therefore appears that the four systems investigated show greater feasibility to be used as qubits in the crystal phase – where they maintain fixed conformational geometries – than in the solution phase, where their conformational behavior is considerably less restrained and their flexible nature renders them unsuitable for use as quantum devices.

Moreover, this study demonstrates how the geometric similarity of molecular conformations, as well as the validity of fitted conformational data, can be evaluated via calculation of the EMD between two conformational sets, giving an insight into the geometric likeness of the two sets. Such analysis can be carried out between any two groups of conformations, provided the geometric structure of each conformation is sufficiently described. The study has further shown how the EMD can also be calculated based on differences in spherical polar angles between conformations, rather than purely based on their separation distances. Such angular EMD analysis is generally likely to be a more valid quantification of conformational similarity as, by definition, conformations fundamentally differ from each other in the degree of rotation about covalent bonds, which is more appropriately described with angles rather than distances. The application of such statistical methods can provide a novel way of comparing two spatial or angular distributions for orientationally selective pulsed dipolar spectroscopy methods – particularly those performed on systems containing multiple centers – beyond comparison of distance distributions, which reduce a geometrical problem to a single dimension.

## Experimental Procedures

### Synthetic Methods

All starting reagents and materials used were sourced from Sigma-Aldrich and/or Alfa. Unless stated otherwise, all reagents and solvents were used without further purification. The syntheses of the hybrid organic–inorganic rotaxanes were carried out in Erlenmeyer Teflon FEP flasks supplied by Fisher. Column chromatography was performed using either 40–63 μm silica from Sigma-Aldrich or a Grace Reverelis X2 Autocolumn with Grace Reverelis NP cartridges. Detailed descriptions of the synthesis of each of the compounds used in this work and intermediates are provided in the Supporting Information.

### Electron Paramagnetic Resonance Methods

CW EPR experiments were performed on a Bruker EMX580 spectrometer. Pulsed EPR experiments were performed on a Bruker ELEXSYS E580 spectrometer. Details of the EPR methods used are provided in the main text above and further information is also provided in the Supporting Information.

### Computational Procedures

Simulations of cw EPR data sets were performed using EasySpin.^43^ Simulations of DEER traces were performed using a home written MATLAB based program, previously described,^13,35,44^ based on EasySpin functions.^43^ Further information is also provided in the Supporting Information. Earth mover’s distance analysis was performed using functions from the MATLAB Central Exchange,^42^ incorporated into home written MATLAB code.

## Data Availability

Raw pulsed EPR data sets are available from the following DOIs: short-tau data set for [**1(2A)**_**3**_] – https://doi.org/10.48420/23268917, long-tau data set for [**1(2A)**_**3**_] – https://doi.org/10.48420/23276132, combined data set for [**1(2A)**_**3**_] – https://doi.org/10.48420/23295149, short-tau data set for [**1(2B)**_**3**_] – https://doi.org/10.48420/23269115, long-tau data set for [**1(2B)**_**3**_] – https://doi.org/10.48420/23276207, combined data set for [**1(2B)**_**3**_] – https://doi.org/10.48420/23295209, data set for [**1(2D)**_**3**_] – https://doi.org/10.48420/23269190, short-tau data set for [**1(2E)**_**3**_] – https://doi.org/10.48420/23269196, long-tau data set for [**1(2E)**_**3**_] – https://doi.org/10.48420/23276246, combined data set for [**1(2E)**_**3**_] – https://doi.org/10.48420/23295236.

## Supporting Information Available

Synthetic methods; crystallography; small angle X-ray scattering; c.w. EPR data; pulsed EPR data; geometric flexibility model; conformational analysis; earth mover’s distance (PDF)

## Notes

Safety Statement: A risk assessment was performed for all reactions and chemicals used in this research. The outcome provided no significant hazards for any chemicals or other substances (such as liquid N2), above standard laboratory procedures. No chemicals used are defined under the Globally Harmonized System (GHS) Classification. However, all {Cr7Ni} compounds should be treated as toxic. No personal protective equipment (PPE) needed beyond standard laboratory procedures are required to perform the reactions defined in this research, and all standard EPR facility safety requirements are proficient for the EPR spectroscopy measurements performed.

## Abbreviations

DEER: double electron–electron resonance
EMD: earth mover’s distance
EPR: electron paramagnetic resonance
MOF: metal–organic framework
SAXS: small-angle X-ray scattering
