# Supplementary material for: Characterizing X-Ray and Solution State Conformations for a Model Qubit System: {Cr7Ni} Ring Rotaxanes on a Mixed Metal Triangle
Source: Inorg Chem. 2024 Nov 17;63(48):22880–91. doi: 10.1021/acs.inorgchem.4c03919 (PMC11615942; doi:10.1021/acs.inorgchem.4c03919)
Supplement: Supplementary file 1 — ic4c03919_si_001.pdf [file ic4c03919_si_001.pdf]

Supporting Information

Characterizing X-ray and Solution State Conformations for a Model Qubit System: {Cr_7Ni} Ring Rotaxanes on a Mixed Metal Triangle

Lubomir Loci,[a,b†] Selena J. Lockyer,[a,b†] Tom S. Bennett,[a] Ciarán J. Rogers,[a,b] Adam Brookfield,[b] Grigore A. Timco,[a] George, F. S. Whitehead,[a] Selina Nawaz,[a] Jack J. Miller,[c,d] Richard E. P. Winpenny[a,b] and Alice M. Bowen[a,b]*

a. Department of Chemistry, The University of Manchester, Oxford Road, Manchester, M13 9PL, The United Kingdom
b. The National Research Facility for Electron Paramagnetic Resonance, The Photon Science Institute, The University of Manchester, Oxford Road, Manchester, M13 9PL, The United Kingdom
c. The MR Research Centre and The PET Research Centre, Aarhus University, Aarhus, Denmark
d. Department of Physics, Clarendon Laboratory, The University of Oxford, Oxford, The United Kingdom

†These authors contributed equally to this work.

*Corresponding author: alice.bowen@manchester.ac.uk

Contents:

Section 1 – Experimental Section……………………………………………………………..S1
Section 2 – Crystallography……………………………………………………………………..S7
Section 3 – Rotaxane Figures……………………………………………………………………..S7
Section 4 – Small Angle X-Ray Scattering……………………………………………………..S10
Section 5 – CW EPR Details……………………………………………………………………..S11
Section 6 – Pulse EPR Details……………………………………………………………………..S15
Section 7 – Geometric Flexibility Model………………………………………………………..S31
Section 8 – Conformational Analysis Figures…………………………………………………S33
Section 9 – Earth Mover’s Distance……………………………………………………………..S38
Section 10 – Angular Earth Mover’s Distance………………………………………………..S40
Section 11 – References……………………………………………………………………………..S42

Section 1 – Experimental Section:

General remarks: All starting reagents and materials used were sourced from Sigma-Aldrich and/or Alfa. Unless stated otherwise, all reagents and solvents were used without further purification. The syntheses of the hybrid organic-inorganic rotaxanes were carried out in Erlenmeyer Teflon® FEP flasks supplied by Fisher. Column chromatography was performed using either 40-63 µm silica from Sigma-Aldrich or a Grace Reverelis ® X2 Autocolumn with Grace Reverelis ® NP cartridges. Chemical shifts are reported in parts per million (ppm) from low to high frequency and referenced to the residual solvent resonance. ESI mass spectrometry and microanalysis were carried out by the services at The University of Manchester.

Synthetic Methods:

1 Heterometallic triangle synthesis [$CrNi_2(\mu_3\text{-}F)(O_2C^tBu)_6(HO_2C^tBu)_3$]:

1.1 Synthesis of **1** [$CrNi_2(\mu_3\text{-}F)(O_2C^tBu)_6(HO_2C^tBu)_3$]:

$^tBuCO2H$ (30 g, 294 mmol), $CrF_3.4H_2O$ (3 g, 16 mmol), $2NiCO_3.3Ni(OH)_2.4H_2O$ (3.90 g, 6.64 mmol) and 2-amino-2-(hydroxymethyl)-1,3-propanediol (1.0 g) were added to a Teflon flask and heated to 140°C for 5 hours. The mixture was left to cool to room temperature, acetonitrile (35 mL) was added, then stirred for 30 minutes. The mixture was filtered and washed with acetonitrile (150 mL), then with cold acetone (15 mL). The solid was dried overnight under N_2. The solid was dissolved in hexane (100 mL), the solution filtered with washing from hexane. The filtrate was evaporated under reduced pressure to produce a dry green crystalline powder. Yield: 3.79 g, 86%. Elemental Analysis %, C: 48.92, H: 7.69, Cr: 4.84, Ni: 10.36.

2 Organic Thread Synthesis ($RR'NH_2$):

Reductive amination - Schiff base condensation method:[S1]

2.1 Synthesis of Thread **A** ($3\text{-}pyC_6H_4CH_2NHCH_2CH_2C_6H_5$):

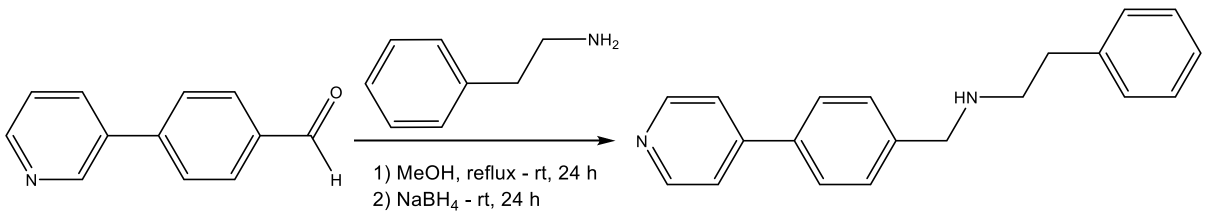

A solution of 4-(3-pyridyl)benzaldehyde (0.55 g, 3 mmol, 1 eq) and phenethylamine (0.36 g, 3 mmol, 1eq) in methanol (50 mL) was refluxed for 5 hours under an N_2 atmosphere, then stirred at room temperature for 3 hours. Excess $NaBH_4$ (0.6 g, 15 mmol, 5 eq) was added and the reaction mixture was stirred for 5 hours. The reaction was quenched with water (50 mL) and the residue was extracted with dichloromethane (3 x 25 mL). The organic extract was dried ($MgSO_4$) and the solvents evaporated under reduced pressure. A clear thin light yellow oil was produced; yield: 0.76 g, 92%. ^1H NMR δ ppm (400 MHz, 293K, $CDCl_3$): 8.40 (dd, 2H), 7.21-7.12 (m, 4H), 7.05 (dd, 2H), 3.67 (s, 2H), 2.83-2.70 (m, 4H), 2.39 (s, 3H); 13C NMR (400 MHz, 293K, CDCl3) δ 149.66 (Ar), 149.20 (Ar),136.98 (Ar), 128.87 (Ar), 126.73 (Ar), 124.22 (Ar), 53.28 (CH2), 49.19 (CH3), 35.69 (CH2) 15.98 (CH3); ESI MS m/z (relative intensity) 289.2 $[M+H]^+$, 311.3 $[M+Na]^+$.

2.2 Synthesis of Thread **B** (3-py$C_6H_4C_6H_4CH_2NHCH_2CH_2C_6H_5$):

3-(4-bromophenyl)pyridine: Prepared using the method previously published:[S2]

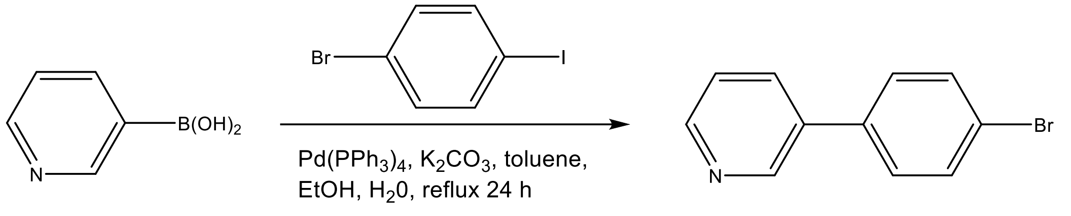

4'-(pyridin-3-yl)-[1,1'-biphenyl]-4-carbaldehyde:

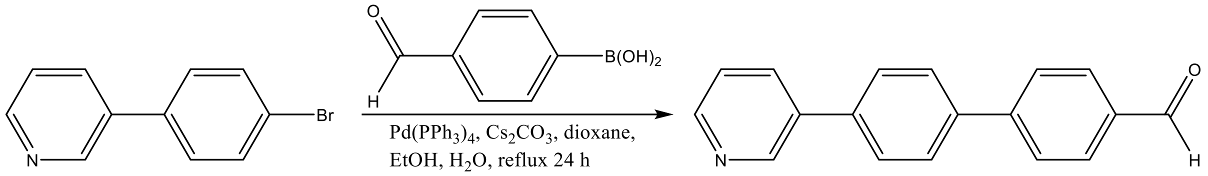

3-(4-bromophenyl)pyridine (3.60 g, 15.38 mmol, 1 eq) and 4-formylphenylboronic acid (2.54 g, 16.92 mmol, 1.1 eq) were dissolved in 200 mL dioxane with stirring. Cesium carbonate (23.55 g, 72.28 mmol, 4.7 eq) was added to the solution with stirring alongside 50 mL ethanol and 40 mL water. The reaction mixture was purged under a N_2 atmosphere for 1 hour before slow addition of tetrakis(triphenylphosphine)palladium(0) (1.78 g, 1.54 mmol, 0.1 eq). The solution was heated to reflux for 24 hours before being allowed to cool to room temperature. The solvent was removed under reduced pressure to leave a brown solid which was dissolved in 150 mL chloroform. The organic layer was washed with water (3 x 50mL) and dried over anhydrous magnesium sulphate and filtered. The solvent was removed under a reduced pressure and the crude product purified by flash column chromatography (gradient elution of $CHCl_3$: EtOAc) to give a white solid (2.94 g, 74 %). ^{1}H NMR δ ppm (400 MHz, 293K, $CDCl_3$): 7.38 – 7.43 (m, 1H), 7.70 – 7.74 (m, 2H), 7.75 – 7.79 (m, 2H), 7.81 (d, 2H), 7.94 (dt, 1H), 7.99 (d, 2H), 8.63 (dd, 1H), 8.92 (d, 1H), 10.08 (s, 1H). ESI MS m/z (relative intensity) 289.2 $[M+H]^+$.

Thread **B**:

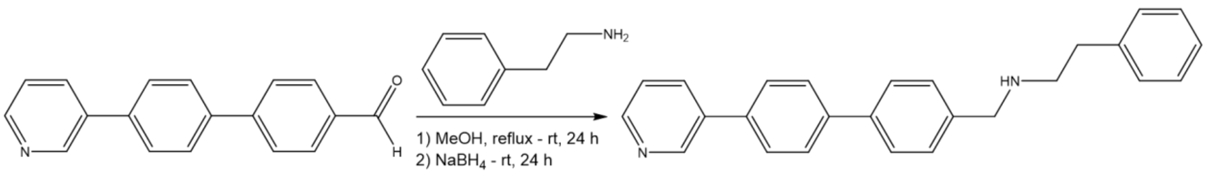

4'-(pyridin-3-yl)-[1,1'-biphenyl]-4-carbaldehyde (2.00 g, 7.71 mmol, 1 eq) was dissolved in 120 mL methanol and purged under a N_2 atmosphere for 1 hour. Phenylethylamine (1.07 mL, 8.48 mmol, 1.1 eq) was added to the solution with stirring and refluxed for 5 hours under an N_2 atmosphere before cooling to room temperature and left to stir overnight. $NaBH_4$ (1.46 g, 38.6 mmol, 5 eq) was added slowly to the solution before stirring overnight to reduce the imine. The reaction was quenched with 70 mL water and solvent removed under a reduced pressure. The resultant solid was dissolved in 150 mL chloroform and washed with water (3 x 50mL) and dried over anhydrous magnesium sulphate and

filtered. The solvent was removed under a reduced pressure before washing the crude product with acetonitrile to yield a white solid (1.72 g, 61 %). ^{1}H NMR δ ppm (400 MHz, 293K, $CDCl_3$): 8.90 (d, 1H), 8.61 (dd, 1H), 7.92 (dt, 1H), 7.70 (m, 2H), 7.66 (m, 2H), 7.60 (m, 2H), 7.36 – 7.42 (m, 3H), 7.27 – 7.32 (m, 2H), 7.19 – 7.25 (m, 3H), 3.88 (s, 2H), 2.96 (t, 2H), 2.88 (t, 2H). ESI MS m/z (relative intensity) 289.2 $[M+H]^+$, 311.3 $[M+Na]^+$.

2.3 Synthesis of Thread **C** ($HOC_6H_4CH_2CH_2NHCH_2C_6H_4SCH_3$):

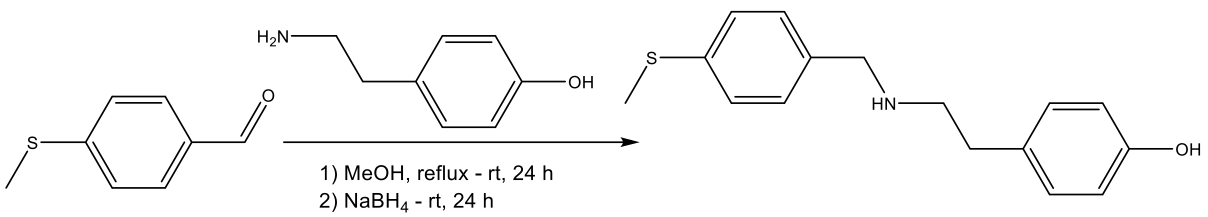

4-(methylthio)benzaldehyde, (3.06 g, 20.12 mmol, 1.1 eq) was dissolved in methanol (150 mL) under a N_2 atmosphere and stirred at room temperature for 1 hour. 4-(2-aminoethyl)phenol, (2.59 g, 18.30 mmol, 1.0 eq) was added under a N_2 atmosphere and the resultant solution refluxed for 5 hours with stirring. The reaction mixture was allowed to cool to room temperature and stirred for a further 20 hours. The solution was cooled to -20°C before the slow addition of $NaBH_4$, (3.46 g, 91.46 mmol, 5.0 eq) over 20 minutes. The solution was allowed to return to room temperature and stirred for an additional 24 hours. The reaction was quenched with water (100 mL) and solvent removed under a reduced pressure. The crude product was dissolved in chloroform (100 mL) and washed with water (3 x 50 mL) and dried over anhydrous magnesium sulphate and filtered. The solvent was removed under a reduced pressure yielding a white solid (3.82 g, 76 %). ^{1}H NMR δ ppm (400 MHz, 293K, DMSO): 7.31 (d, 2H), 7.25 (d, 2H), 7.03 (d, 2H), 6.72 (d, 2H), 3.71 (s, 2H), 2.67 (t, 2H), 2.57 (t, 2H), 2.51 (s, 3H). ESI MS m/z (relative intensity) 289.2 $[M+H]^+$, 311.3 $[M+Na]^+$.

3 Heterometallic Rotaxane Synthesis ($(RR'NH_2)[Cr_7NiF_8(O_2C^tBu)_{16}]$):

3.1 Synthesis of **2A** (**A**H)$[Cr_7NiF_8(O_2C^tBu)_{16}]$:

$^tBuCO_2H$ (20 g, 195 mmol, 70 eq), $CrF_3.4H_2O$ (3 g, 16 mmol, 7 eq), Thread **A** (0.8 g, 2.78, 1 eq) were added to a Teflon flask and heated to 140°C for 30 minutes. $2NiCO_3.3Ni(OH)_2.4H_2O$ (0.35 g, 0.6 mmol, 0.2 eq) was added and the melt heated at 140°C for a further 60 minutes. Then the heat was then increased to 160°C for 20 hours. The mixture was left to cool to room temperature, 35 mL acetonitrile was added, stirred for 1 hour, then filtered. The isolated green powder was washed with acetonitrile. Column chromatography (20:1 toluene:ethyl acetate) produced three fractions, the third fraction containing the product. Solvents were removed to produce a dry green powder. Yield: 1.52 g, 26%. Elemental analysis (%) calcd. for $C_{100}H_{165}Cr_7F_8N_2NiO_{32}$: C 48.3, H 6.74, Cr 14.66, N 1.13, Ni 2.36; found: C 49.46, H 6.76, Cr 13.32, N 1.15, Ni 2.25. ESI MS m/z (relative intensity) 2483 $[M+H]^+$, 2505 $[M+Na]^+$

3.2 Synthesis of **2B** (**B**H)$[Cr_7NiF_8(O_2C^tBu)_{16}]$:

$^tBuCO_2H$ (30 g, 297 mmol, 127 eq) and Thread **B** (0.86 g, 2.33 mmol, 1 eq) was dissolved in an open Teflon flask at 140°C before adding $CrF_3.4H_2O$ (3.00 g, 16.57 mmol, 7.1 eq) and stirring at 140°C for 30 minutes. $[Ni_2(H_2O)(O_2CCMe_2)_4(HO_2CCMe_2)_4]$ (0.6 g, 0.633 mmol, 0.25 eq) was then added slowly and the solution heated to 160°C for 24 hours. The mixture was left to cool to room temperature, 35 mL acetonitrile was added, stirred for 1 hour, then filtered. The isolated green powder was washed with

acetonitrile. The product was extracted in toluene (100 mL) and solvent removed under a reduced pressure. The crude product purified by flash column chromatography (DCM: EtOAc, 9:1) to give a green crystalline solid (2.07 g, 19 %). Elemental analysis (%) calcd. for $C_{106}H_{169}Cr_7F_8N_2NiO_{32}$: C 49.77, H 6.66, Cr 14.23, N 1.10, Ni 2.29; found: C 49.77, H 6.66, Cr 13.13, N 1.04, Ni 2.23. ESI MS m/z (relative intensity) 2559 [M+H]+, 2581 [M+Na]+.

3.3 Synthesis of **2C** (**C**H)$[Cr_7NiF_8(O_2C^tBu)_{16}]$:

$^tBuCO_2H$ (30 g, 297 mmol, 127 eq) and Thread **C** (1.91 g, 7.00 mmol, 3.0 eq) was dissolved in an open Teflon flask at 140°C until completely dissolved. $CrF_3\cdot 4H_2O$ (3.00 g, 16.57 mmol, 7.1 eq) and $[Ni_2(H_2O)(O_2CCMe_2)_4(HO_2CCMe_2)_4]$ (1.33 g, 1.40 mmol, 0.6 eq) was added to the solution and stirred for 24 hours. The mixture was left to cool to room temperature, 100 mL acetonitrile was added, stirred for 1 hour, then filtered. The product was extracted in toluene (100 mL) and solvent removed under a reduced pressure. The crude product purified by flash column chromatography ($CHCl_3$: EtOAc, 9:1) to give a green crystalline solid (0.85 g, 15 %). Elemental analysis (%) calcd. for $C_{96}H_{164}SCr_7F_8NNiO_{33}$: C 46.76, H 6.66, N 0.57, S 1.30, Cr 14.76, Ni 2.38; found: C 46.95, H 6.73, N 0.57, S 1.00, Cr 14.09, Ni 2.42. ESI MS m/z (relative intensity) 2467.6 $[M+H]^+$, 2489.6 $[M+Na]^+$.

3.4 Synthesis of **2D** (**D**H)$[Cr_7NiF_8(O_2C^tBu)_{16}]$:

Pyrimidine-5-carboxylic acid (0.2 g, 1.62 mmol, 4 eq), dicyclohexylcarbodiimide (0.34 g, 1.62 mmol 4 eq) and 4-dimethylaminopyridine (0.15 g, 1.62 mmol, 4 eq) was stirred in DCM (35 mL) at 30°C until all dissolved. **2C** (1 g, 0.41 mmol, 1 eq) was then added to the solution, which was continued to be stirred and heated at 30°C for 24 hours. The crude product purified by flash column chromatography ($CHCl_3$: EtOAc, 10:1) to give a green crystalline solid (0.52 g, 50 %). Elemental analysis (%) calcd. for $C_{96}H_{164}SCr_7F_8NNiO_{33}$: C 46.76, H 6.66, N 0.57, S 1.30, Cr 14.76, Ni 2.38; found: C 46.95, H 6.73, N 0.57, S 1.00, Cr 14.09, Ni 2.42. ESI MS m/z (relative intensity) 2467.6 $[M+H]^+$, 2489.6 $[M+Na]^+$.

3.5 Synthesis of **2E** (**E**H)$[Cr_7NiF_8(O_2C^tBu)_{16}]$:

Nicotinic acid (0.048 g, 0.34 mmol, 4 eq), dicyclohexylcarbodiimide (0.080 g, 0.34 mmol 4 eq) and 4-dimethylaminopyridine (0.048 g, 0.34 mmol, 4 eq) was stirred in DCM (20 mL) at 30°C until all dissolved. **2C** (0.24 g, 0.097 mmol, 1 eq) was then added to the solution, which was continued to be stirred and heated at 30°C for 24 hours. The crude product purified by flash column chromatography ($CHCl_3$: EtOAc, 10:1) to give a green crystalline solid (0.15 g, 60 %). Elemental analysis (%) calcd. for $C_{96}H_{164}SCr_7F_8NNiO_{33}$: C 46.76, H 6.66, N 0.57, S 1.30, Cr 14.76, Ni 2.38; found: C 46.95, H 6.73, N 0.57, S 1.00, Cr 14.09, Ni 2.42. ESI MS m/z (relative intensity) 2467.6 $[M+H]^+$, 2489.6 $[M+Na]^+$.

4 Hybrid heterometallic rotaxanes and triangles synthesis $[CrNi_2(\mu_3\text{-}F)(O_2C^tBu)_6]\{(RR'NH_2)[Cr_7NiF_8(O_2C^tBu)_{16}]\}_3$:

4.1 Synthesis of [**1**(**2A**)$_3$] $[CrNi_2(\mu_3\text{-}F)(O_2C^tBu)_6]\{$(**A**H)$[Cr_7NiF_8(O_2C^tBu)_{16}]\}_3$:

To a warm solution (40 °C) of **2A** (0.5 g, 0.20 mmol, 3 eq) in THF (20 mL), **1** (0.075 g, 0.068 mmol, 1 eq) was added and the solution stirred for 10 minutes, filtered and once cooled to room temperature acetonitrile (5 mL) was added to the solution. Green crystals suitable for single crystal X-ray diffraction were grown via slow evaporation of the solvents over 48 hours. The crystals were separated by

filtration and washed with THF, then acetonitrile. Yield: 0.20 g (35%). Elemental analysis %: calc. for $C_{330}H_{549}Cr_{22}F_{25}N_6Ni_5O_{108}$: Cr 13.88, Ni 3.56, C 48.09, H 6.71, N 1.02; found: Cr 11.89, Ni 3.09, C 46.97, H 6.55, N 0.80.

4.2 Synthesis of [**1**(**2B**)$_3$] $[CrNi_2(\mu_3\text{-}F)(O_2C^tBu)_6]\{(\mathbf{B}H)[Cr_7NiF_8(O_2C^tBu)_{16}]\}_3$:

To a hot solution (50 °C) of **2B** (0.100 g, 0.039 mmol) in acetone (5 mL), **1** (0.015 g, 0.013 mmol) was added and the solution stirred for 10 minutes, filtered and allowed to cool to room temperature. Green crystals suitable for single crystal X-ray diffraction were grown via slow evaporation of the solvents over 48 hours. The crystals were separated by filtration and washed with acetone, then acetonitrile. Yield: 0.068 g (61%). Elemental analysis %: calc. for C315H543Cr22F25N6Ni5O108S3: Cr 14.03, Ni 3.60, C 46.42, H 6.71, S 1.18, N 1.03; found: Cr 13.32, Ni 4.44, C 45.19, H 6.76, S 1.05, N 1.01.

4.3 Synthesis of [**1**(**2D**)$_3$] $[CrNi_2(\mu_3\text{-}F)(O_2C^tBu)_6]\{(\mathbf{D}H)[Cr_7NiF_8(O_2C^tBu)_{16}]\}_3$:

To a hot solution (50 °C) of **2D** (0.075 g, 0.029 mmol) in acetone (15 mL), **1** (0.011 g, 0.010 mmol) was added and the solution stirred for 10 minutes, filtered and allowed to cool to room temperature. Green crystals suitable for single crystal X-ray diffraction were grown via slow evaporation of the solvents over 48 hours. The crystals were separated by filtration and washed with acetone, then acetonitrile. Yield: 0.058 g (71%). Elemental analysis %: calc. for $C_{333}H_{552}Cr_{22}F_{25}N_9Ni_5O_{114}S_3$: Cr 13.43, Ni 3.45, C 46.97, H 6.53, S 1.13, N 1.48; found: Cr 12.03, Ni 3.38, C 47.08, H 6.81, S 0.95, N 1.97.

4.4 Synthesis of [**1**(**2E**)$_3$] $[CrNi_2(\mu_3\text{-}F)(O_2C^tBu)_6]\{(\mathbf{E}H)[Cr_7NiF_8(O_2C^tBu)_{16}]\}_3$:

To a hot solution (50 °C) of **2E** (0.075 g, 0.029 mmol) in acetone (15 mL), **1** (0.011 g, 0.010 mmol) was added and the solution stirred for 10 minutes, filtered and allowed to cool to room temperature. Green crystals suitable for single crystal X-ray diffraction were grown via slow evaporation of the solvents over 48 hours. The crystals were separated by filtration and washed with acetone, then acetonitrile. Yield: 0.040 g (47%). Elemental analysis %: calc. for $C_{336}H_{555}Cr_{22}F_{25}N_6Ni_5O_{114}S_3$: Cr 13.44, Ni 3.45, C 47.41, H 6.57, S 1.13, N 0.99; found: Cr 12.65, Ni 3.49, C 47.31, H 6.70, S 1.04, N 1.78.

Section 2 – Crystallography:

Data Collection:

X-Ray data for compounds [**1**(**2N**)$_3$] (where **N** is **A**, **B**, **D**, or **E**) were collected using a Rigaku FR-X with Cu-Kα radiation equipped with a HypixHE6000 detector, equipped with an Oxford Cryosystems nitrogen flow gas system. Compounds [**1**(**2A**)$_3$] and [**1**(**2B**)$_3$] were measured at 150 K as the lowest temperature achievable without crystal degradation, possibly due to a phase transition. Compound [**1**(**2D**)$_3$] was measured at 100 K, which is the base temperature of the instrument. Compound [**1**(**2E**)$_3$] showed a clear phase transition and had to be measured at 235 K. Data was measured using CrysAlisPro suite of programs. CCDC 2310806, CCDC 2310807, CCDC 2310808, and CCDC 2310809 contain the supplementary crystallographic data for these compounds respectively; these can be obtained free of charge via www.ccdc.cam.ac.uk/structures/? (or from the Cambridge Crystallographic Data Centre, 12 Union Road, Cambridge CB2 1EZ, UK; phone: +44 (0)1223 336408; or deposit@ccdc.cam.ac.uk).

Section 3 – Rotaxane Figures:

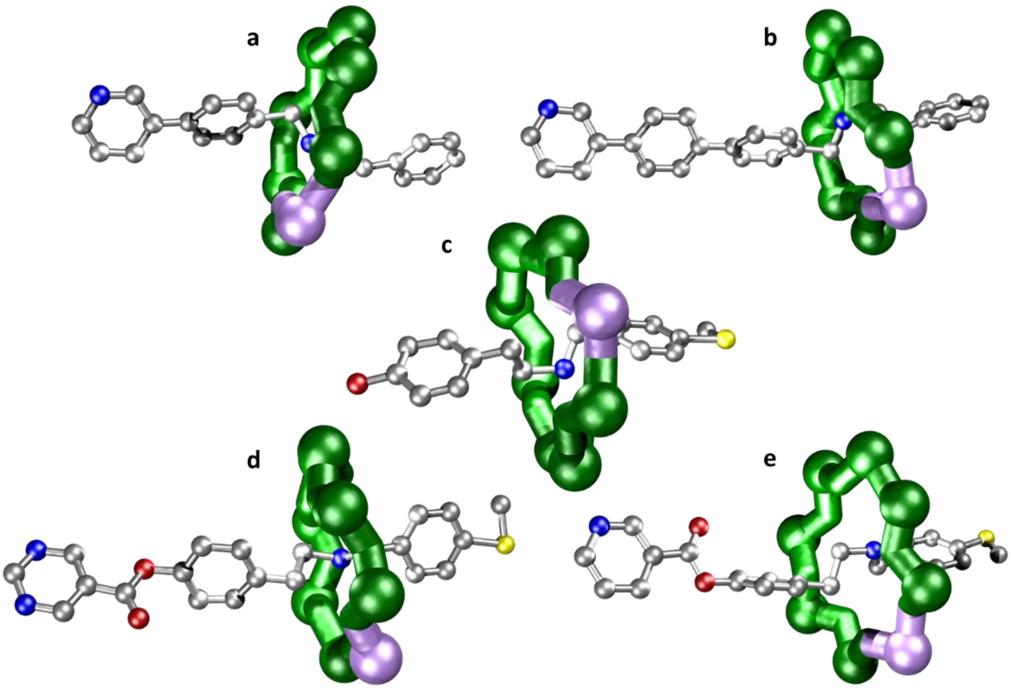

Figure S1: Schematic structure of [2]rotaxanes a) **2A**, b) **2B**, c) **2C**, d) **2D**, and e) **2E**. Atom colors, as per main text.

Crystal Packing Figures:

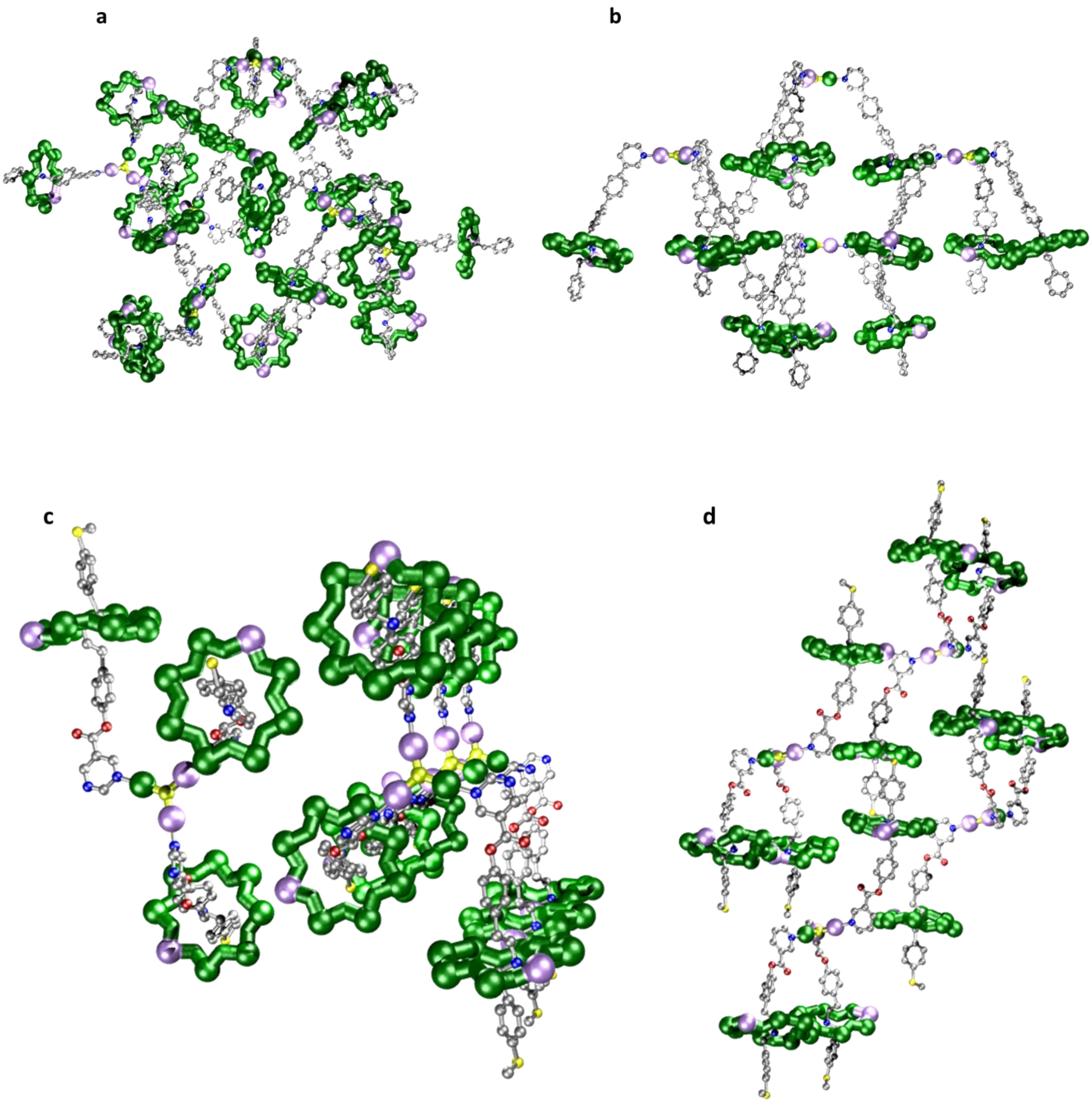

Figure S2: Crystal packing for compounds a) [**1**(**2A**)$_3$], b) [**1**(**2B**)$_3$], c) [**1**(**2D**)$_3$], and d) [**1**(**2E**)$_3$]. Atom colors, as per main text.

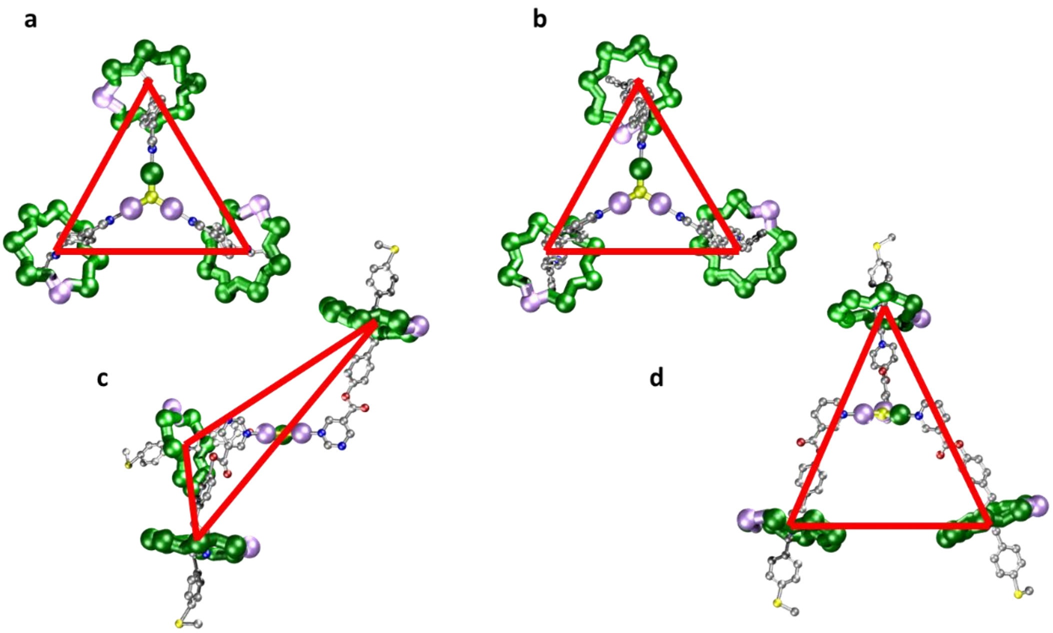

Figure S3: Visualizations of the triangle geometry formed by the center of each {Cr_7Ni} ring for compounds a) [**1**(**2A**)$_3$], b) [**1**(**2B**)$_3$], c) [**1**(**2D**)$_3$], and d) [**1**(**2E**)$_3$]. Atom colors, as per main text.

Section 4 – Small Angle X-Ray Scattering:

Small Angle X-ray Scattering (SAXS) data were obtained on compounds [**1**(**2A**)$_3$] and [**1**(**2E**)$_3$] (Figure S4) and compared with predicted SAXS data from a calculation based on the crystal structures of [**1**(**2N**)$_3$] where **N** = {**A**, **B**, **D**, **E**}. Firstly, the two experimental traces for [**1**(**2A**)$_3$] and [**1**(**2E**)$_3$] contain two distributions centered at *ca.* 8 and 24 Å, and they are not hugely shifted from the distance that would be predicted from the crystal structures. We conclude that the connectivity is maintained in solution. In addition, the pair-pair distribution functions (PPDFs) are noticeably broader than those predicted from the crystal structure, and in both [**1**(**2A**)$_3$] and [**1**(**2E**)$_3$] extend to 30 Å, beyond the longest distances we would predict from the crystal structures. This suggests that the structures are flexible in solution, which we have seen in a related complex where only two [2]rotaxanes are attached to a central triangle.[S3] Comparing the measured SAXS data to the inter-ring metal-metal distance distributions obtained from the best-fit conformations to the DEER data, the distributions for both [**1**(**2A**)$_3$] and [**1**(**2E**)$_3$] extend to just above 30 Å (Figures 4b and S13b) in good agreement with the experimental SAXS data.

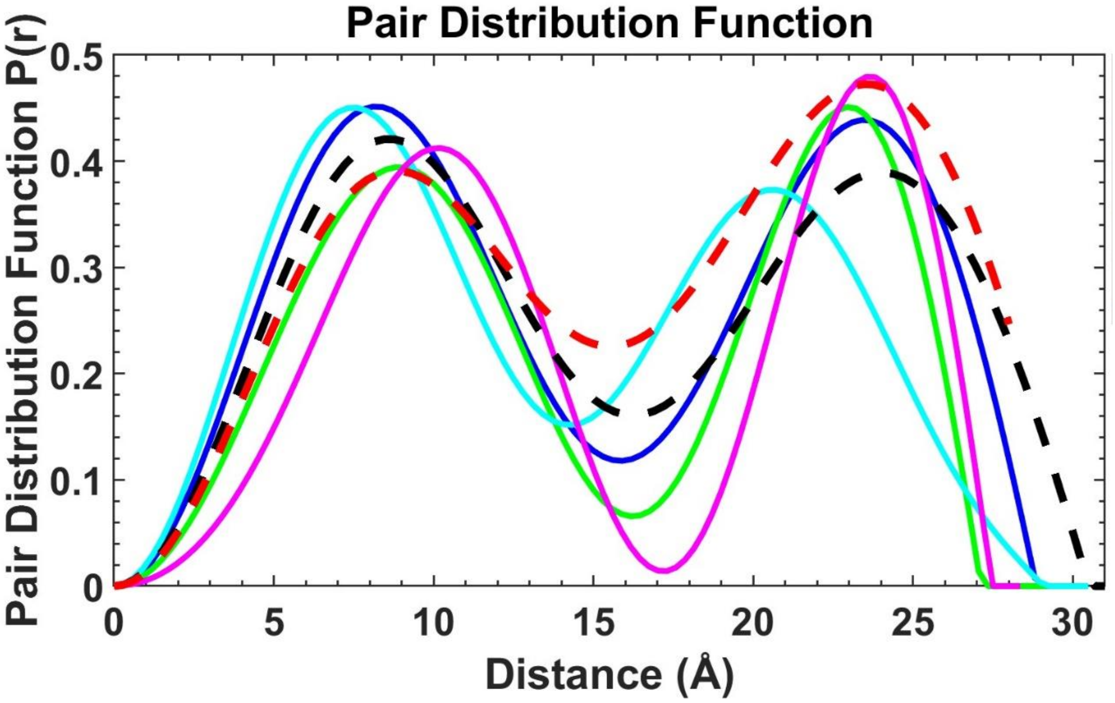

Figure S4. SAXS data presented as PPDFs, measured for [**1**(**2A**)$_3$] (dashed black line) and [**1**(**2E**)$_3$] (dashed red), and calculated from crystal structures for [**1**(**2A**)$_3$] (dark blue), [**1**(**2B**)$_3$] (green), [**1**(**2D**)$_3$] (cyan), and [**1**(**2E**)$_3$] (magenta).

We attempted to achieve a better fit to measured data by performing atomistic molecular dynamic simulations on these structures. However, the simulations never converged, indicating a very large number of potential conformations is present in solution; this is consistent with results obtained from DEER spectroscopy (see main paper).

Section 5 – CW EPR Details:

Continuous-wave Q-band (*ca.* 34 GHz) EPR spectra were recorded with a Bruker EMX580 spectrometer. The continuous-wave data were collected on polycrystalline powders and a solution of 1:1 toluene / DCM at 5 K (unless otherwise stated) using liquid helium cooling. All continuous-wave spectra were field corrected using a 'Strong Pitch' standard (g = 2.0028) and all powder samples were checked for any polycrystalline nature, by measuring multiple random rotations. Spectral simulations were performed using the EasySpin 5.2.30 simulation software[S4] unless otherwise stated.

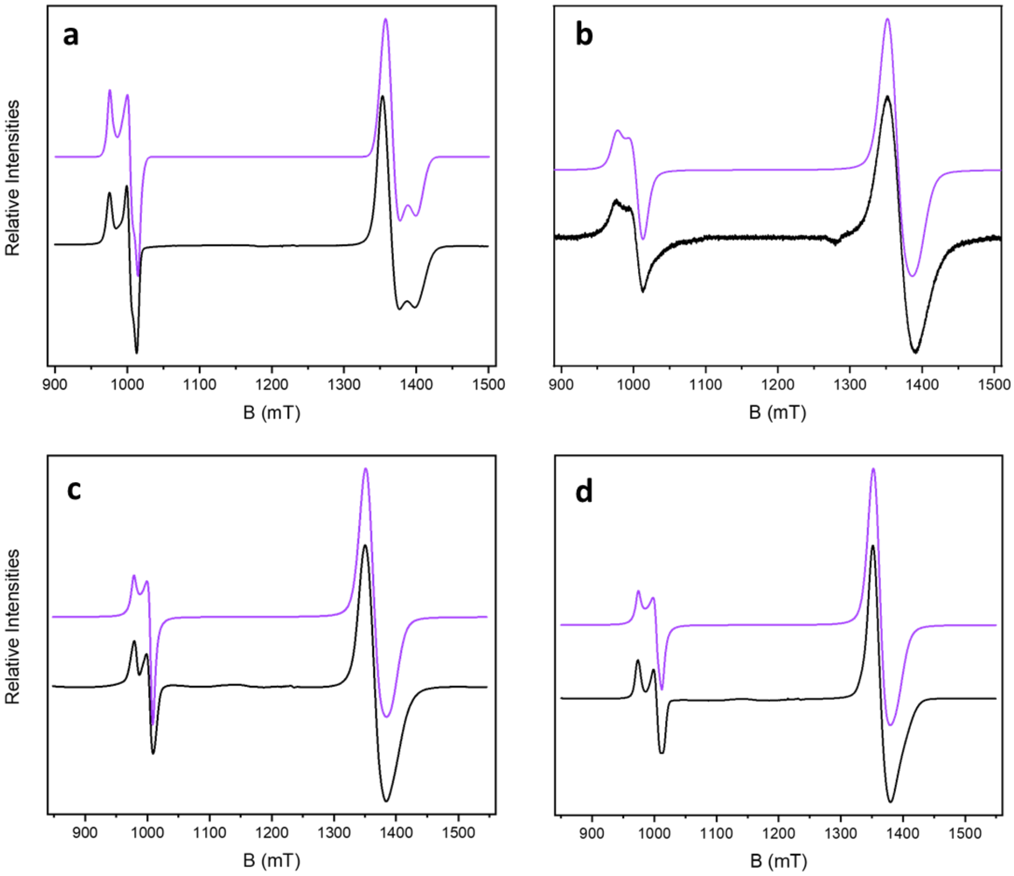

Figure S5: CW (ca. 34 GHz) EPR spectra of [**1**(**2A**)$_3$] (a), [**1**(**2B**)$_3$] (b), [**1**(**2D**)$_3$] (c), and [**1**(**2E**)$_3$] (d) as a powder at 5 K (black) and simulation (purple). Experimental frequencies for [**1**(**2A**)$_3$], [**1**(**2B**)$_3$], [**1**(**2D**)$_3$] and [**1**(**2E**)$_3$]: 34.032794 GHz, 34.035351 GHz, 34.103794 GHz, and 34.023294 GHz respectively.

Table S1: Easyspin simulation parameters for powder EPR samples

**Compound**	g_{xyz} **{Cr$_7$Ni}**	**gStrain xyz Ring**	g_{xyz} **{CrNi$_2$}**	**Linewidth (mT)**
[**1**(**2A**)$_3$] - Pwd	1.785<br>1.785<br>1.735	0.024<br>0.024<br>0.024	2.422<br>2.395<br>2.492	5 Gauss
[**1**(**2B**)$_3$] - Pwd	1.785<br>1.785<br>1.745	0.030<br>0.030<br>0.030	2.420<br>2.400<br>2.490*<br>gStrain iso 0.030	10 Lorz
[**1**(**2D**)$_3$] - Pwd	1.795<br>1.795<br>1.750	0.030<br>0.030<br>0.030	2.423<br>2.415<br>2.490	10 Lorz
[**1**(**2E**)$_3$] - Pwd	1.790<br>1.790<br>1.750	0.025<br>0.025<br>0.030	2.423<br>2.400<br>2.495	10 Lorz

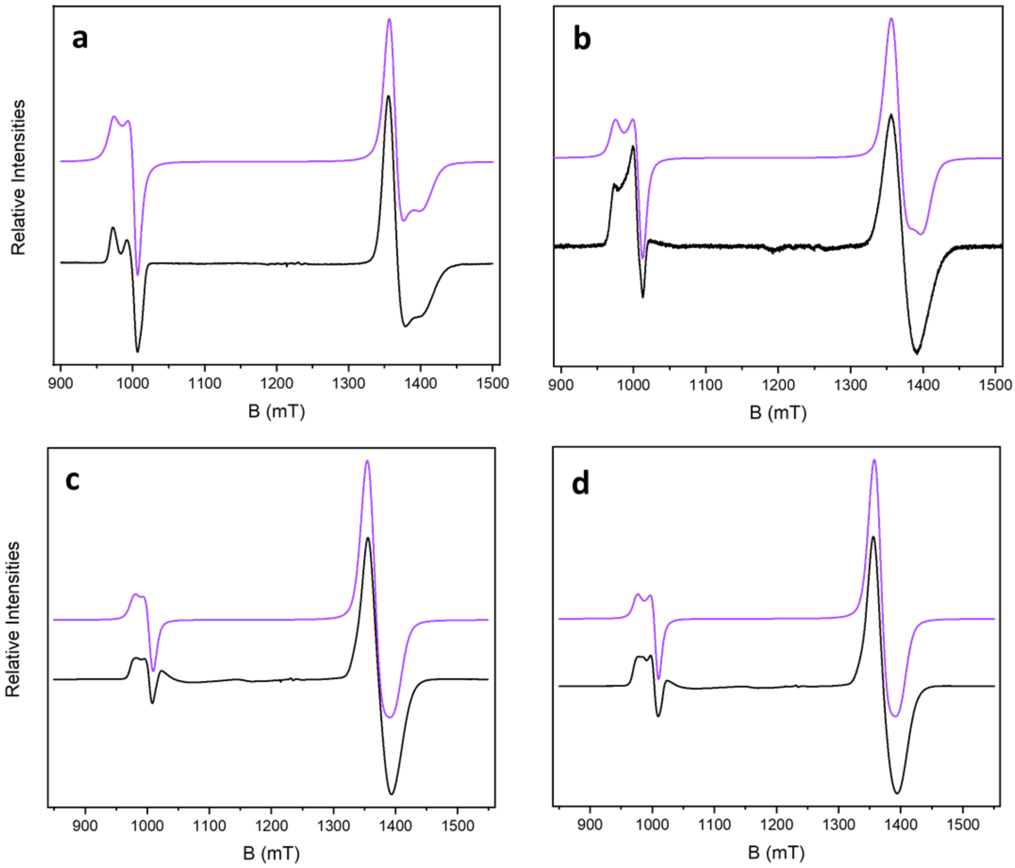

Figure S6a: CW (ca. 34 GHz) EPR spectra of [**1**(**2A**)$_3$] (a), [**1**(**2B**)$_3$] (b), [**1**(**2D**)$_3$] (c), and [**1**(**2E**)$_3$] (d) as a solution at 5 K (black) and simulation (purple). Experimental frequencies for [**1**(**2A**)$_3$], [**1**(**2B**)$_3$], [**1**(**2D**)$_3$], and [**1**(**2E**)$_3$]: 34.009638 GHz, 34.021614 GHz, 34.035111GHz, and 33.999886 GHz respectively.

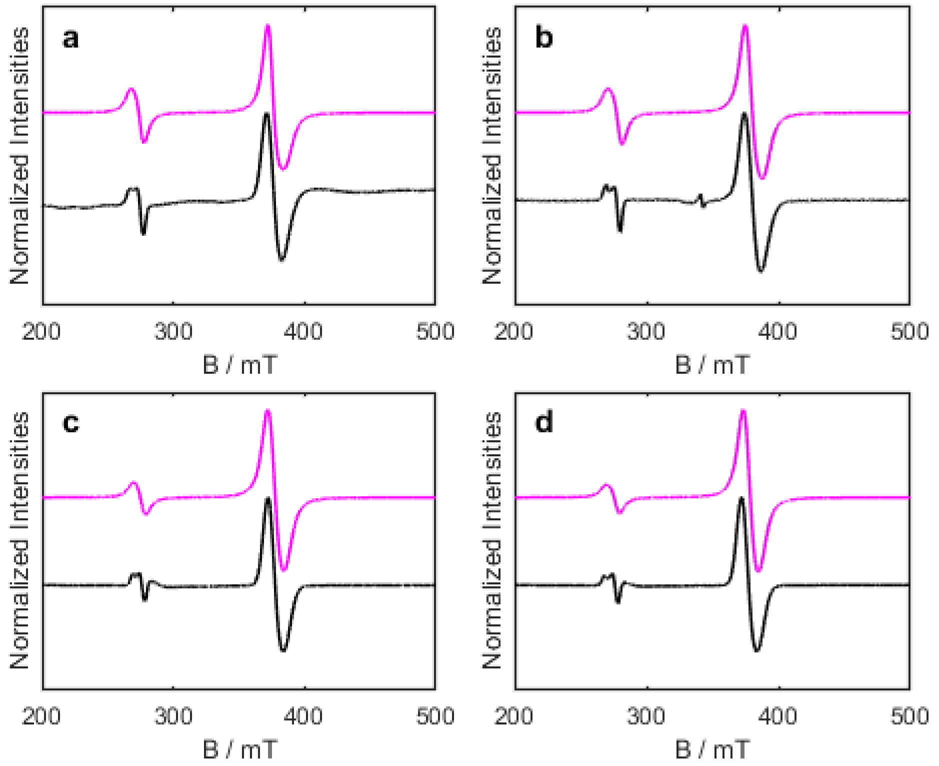

Figure S6b. X-band CW (ca. 9.4 GHz) EPR spectra of [**1**(**2A**)$_3$] (a), [**1**(**2B**)$_3$] (b), [**1**(**2D**)$_3$] (c), and [**1**(**2E**)$_3$] (d) as a solution at 5 K (black) and simulation (purple). Experimental frequencies for [**1**(**2A**)$_3$], [**1**(**2B**)$_3$], [**1**(**2D**)$_3$], and [**1**(**2E**)$_3$]: 9.3466 GHz, 9.4230 GHz, 9.3722 GHz, and 9.3756 GHz respectively.

Table S2: Easyspin simulation parameters for frozen solution EPR samples

**Compound**	**g_{xyz} {Cr_7Ni}**	**gStrain xyz Ring**	**g_{xyz} {$CrNi_2$}**	**gStrain xyz Tri**	**Linewidth (mT)**
[**1**(**2A**)$_3$] - Sol	1.785<br>1.785<br>1.730	0.015<br>0.015<br>0.03	2.425<br>2.415<br>2.498	N/A	10 Lorz
[**1**(**2B**)$_3$] - Sol	1.785<br>1.785<br>1.730	0.025<br>0.025<br>0.025	2.410<br>2.405<br>2.495	0.020<br>0.020<br>0.025	10 Lorz
[**1**(**2D**)$_3$] - Sol	1.787<br>1.787<br>1.738	0.030<br>0.030<br>0.030	2.420<br>2.415<br>2.485	0.025<br>0.025<br>0.030	10 Lorz
[**1**(**2E**)$_3$] - Sol	1.782<br>1.782<br>1.737	0.027<br>0.027<br>0.027	2.412<br>2.412<br>2.490	0.020<br>0.020<br>0.025	10 Lorz

Section 6 – Pulse EPR Details:

Compound [**1**(**2A**)$_3$]:

Identity confirmation:		*See XRD data (and DEER)*
Sample conditions:	Concentration	0.2 mM
	Tube diameter	3 mm
	Sample volume	0.1-0.2 ml
	Cryoprotectant	Liquid N_2
	Freezing procedure	Flash-freezing
	Deuteration	*None*
	Solvent	Toluene
	Temperature	3 K
Instrumentation:	Spectrometer	Bruker Elexsys E580
	Resonator	MS3 (split-ring)
EPR Parameters:	Pump π-pulse length	10 ns
	Det π/2-pulse length	10 ns
	Det π-pulse length	20 ns
	Pump frequency	9.25 GHz
	Det frequency	9.40 GHz
	Pump-det offset	-150 MHz
	Pump pulse shape	Rectangular
	Det pulse shape	Rectangular
	τ_1 length	120 ns
	τ_2 length	*See next page*
	Shot repetition time	1020 µs
	Time increment	4 ns
	Accumulation time	*See next page*
	Number of averages	*See next page*
Nuclear modulation averaging procedure:	Tau-averaging (τ_1, ^{1}H)	Time step: 8 ns<br>Number of τ_1 to average: 8
Measures to reduce multi-spin effects:		*None as multi-spin effects assumed to be negligible at X-band (low inversion efficiencies)*
Data parameters:	Modulation depth	*See next page*
	SNR (wrt mod depth)	*See next page*
	Zero-time offset	80 ns

τ_2 = 800 ns	**3690 G**	**3730 G**	**3770 G**	**3810 G**	**3850 G**
Accumulation time / hours	9.5	3	3	3	4.5
Number of scans	50	15	15	15	25
Shots per point	20	20	20	20	20
Modulation depth / %	20.7	25.9	21.3	12.5	5.0
SNR wrt MD* / no units	83	234	476	162	81

τ_2 = 1100 ns	**3690 G**	**3730 G**	**3770 G**	**3810 G**	**3850 G**
Accumulation time / hours	43	11	11	11	35.5
Number of scans	175	45	45	45	145
Shots per point	20	20	20	20	20

*calculated using the SnrCalculator program[S5]

Table S3. Normalized raw experimental DEER traces (blue) of [**1**(**2A**)$_3$] at two different τ_2 values prior to background correction, overlaid with the corresponding background (dashed red) modelled as a homogeneous (exponential) decay.

**[1(2A)$_3$]**	$\tau_2 = 800\ ns$	$\tau_2 = 1100\ ns$
B = 3690 G	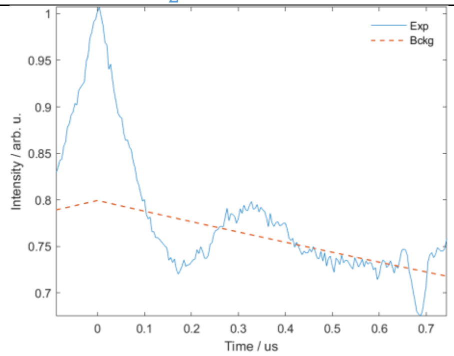	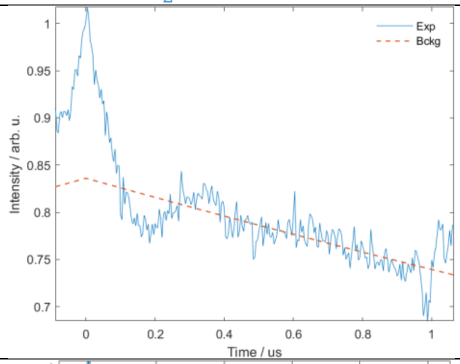
B = 3730 G	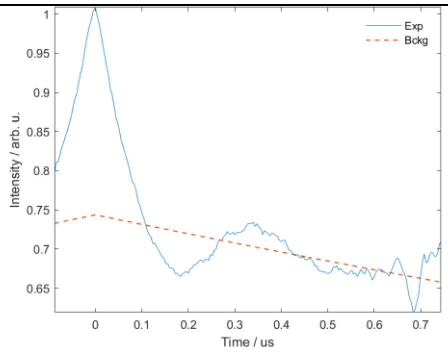	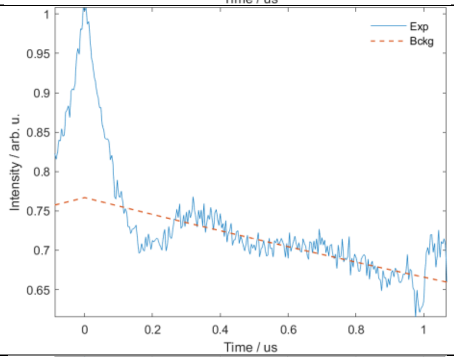
B = 3770 G	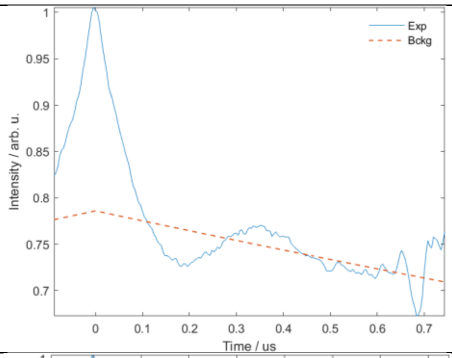	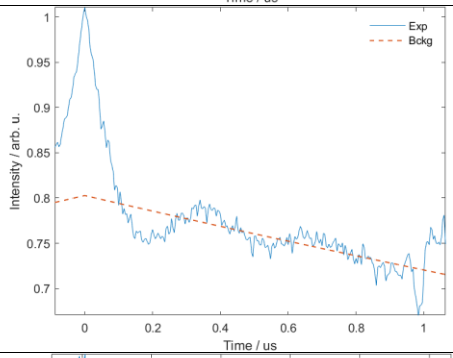
B = 3810 G	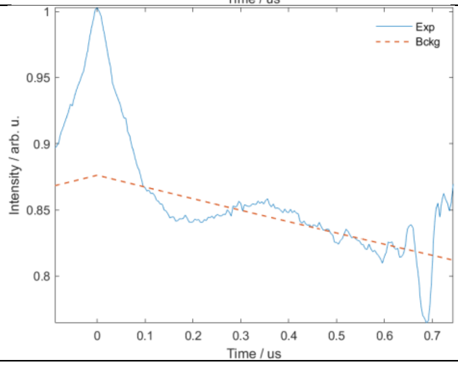	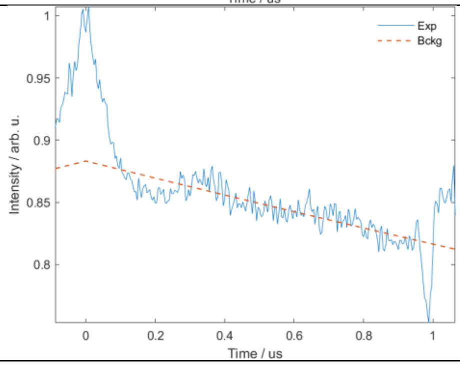

B = 3850 G	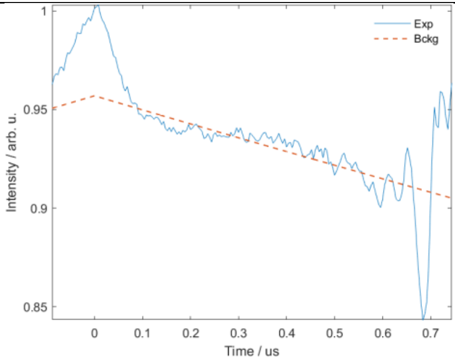 Exp<br>Bckg<br>Intensity / arb. u.<br>Time / us	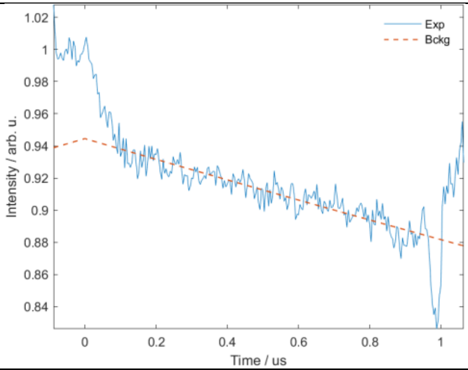 Exp<br>Bckg<br>Intensity / arb. u.<br>Time / us

Compound [**1**(**2B**)$_3$]:

Identity confirmation:		*See XRD data (and DEER)*
Sample conditions:	Concentration	0.2 mM
	Tube diameter	3 mm
	Sample volume	0.1-0.2 ml
	Cryoprotectant	Liquid N_2
	Freezing procedure	Flash-freezing
	Deuteration	*None*
	Solvent	Toluene
	Temperature	3 K
Instrumentation:	Spectrometer	Bruker Elexsys E580
	Resonator	MS3 (split-ring)
EPR Parameters:	Pump π-pulse length	10 ns
	Det π/2-pulse length	10 ns
	Det π-pulse length	20 ns
	Pump frequency	9.26 GHz
	Det frequency	9.41 GHz
	Pump-det offset	-150 MHz
	Pump pulse shape	Rectangular
	Det pulse shape	Rectangular
	τ_1 length	120 ns
	τ_2 length	*See next page*
	Shot repetition time	1020 µs
	Time increment	4 ns
	Accumulation time	*See next page*
	Number of averages	*See next page*
Nuclear modulation averaging procedure:	Tau-averaging (τ_1, ^{1}H)	Time step: 8 ns<br>Number of τ_1 to average: 8
Measures to reduce multi-spin effects:		*None as multi-spin effects assumed to be negligible at X-band (low inversion efficiencies)*
Data parameters:	Modulation depth	*See next page*
	SNR (wrt mod depth)	*See next page*
	Zero-time offset	80 ns

τ_2 = 800 ns	**3690 G**	**3730 G**	**3770 G**	**3810 G**	**3850 G**
Accumulation time / hours	5.5	3	2	3	5.5
Number of scans	30	15	10	15	30
Shots per point	20	20	20	20	20
Modulation depth / %	14.6	18.9	15.2	9.0	3.6
SNR wrt MD* / no units	39	112	144	63	25

τ_2 = 1100 ns	**3690 G**	**3730 G**	**3770 G**	**3810 G**	**3850 G**
Accumulation time / hours	34.5	17	10	17	34.5
Number of scans	140	70	40	70	140
Shots per point	20	20	20	20	20

*calculated using the SnrCalculator program[S5]

Table S4. Normalized raw experimental DEER traces (blue) of [**1**(**2B**)$_3$] at two different τ_2 values prior to background correction, overlaid with the corresponding background (dashed red) modelled as a homogeneous (exponential) decay.

**[1(2B)$_3$]**	$\tau_2 = 800\ ns$	$\tau_2 = 1100\ ns$
B = 3690 G	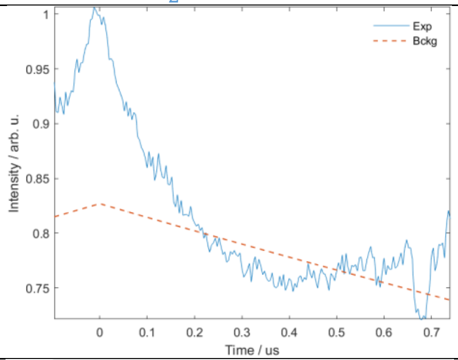	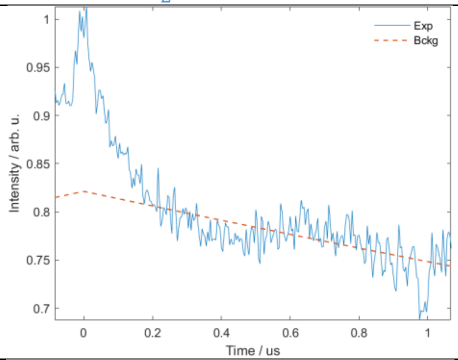
B = 3730 G	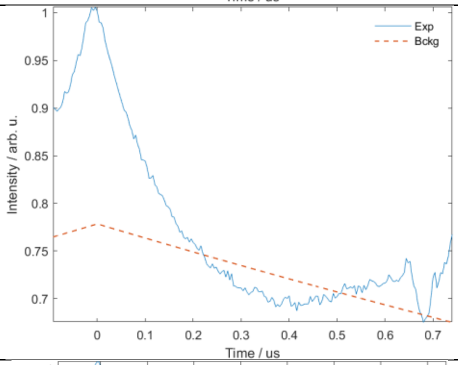	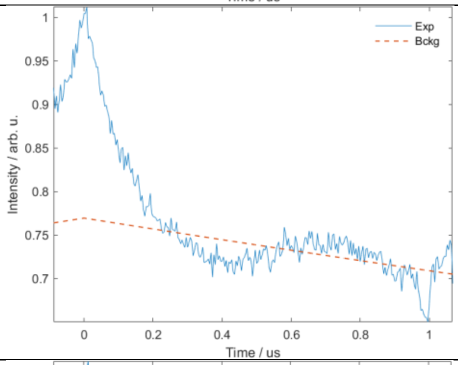
B = 3770 G	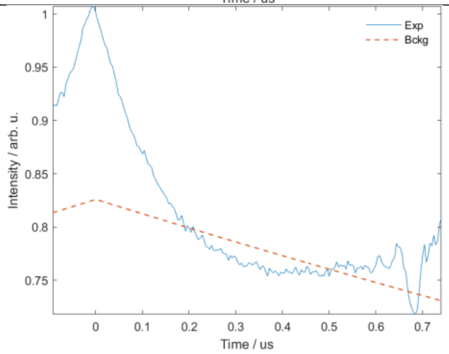	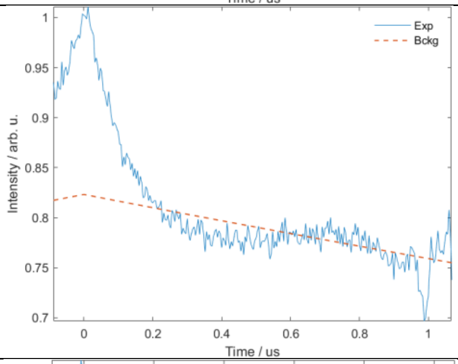
B = 3810 G	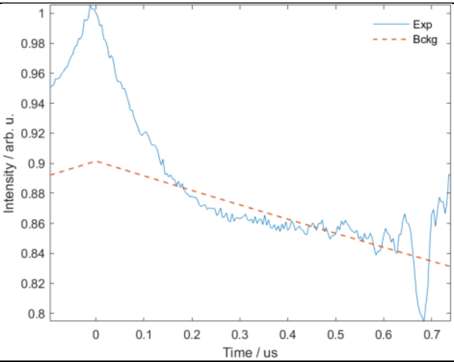	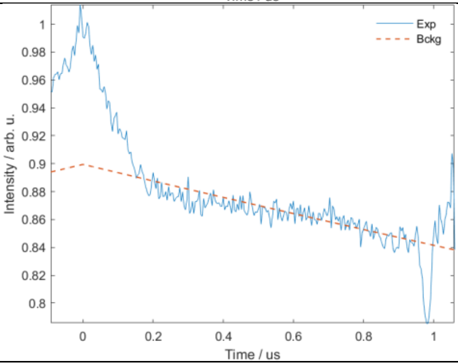

B = 3850 G	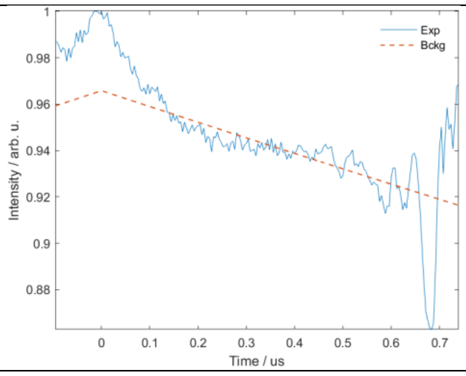<br>Exp<br>Bckg<br>Intensity / arb. u.<br>1<br>0.98<br>0.96<br>0.94<br>0.92<br>0.9<br>0.88<br>0<br>0.1<br>0.2<br>0.3<br>0.4<br>0.5<br>0.6<br>0.7<br>Time / us	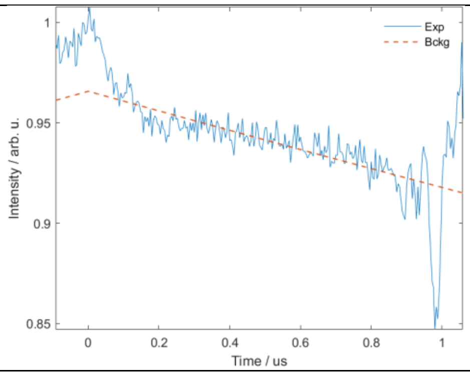<br>Exp<br>Bckg<br>Intensity / arb. u.<br>1<br>0.95<br>0.9<br>0.85<br>0<br>0.2<br>0.4<br>0.6<br>0.8<br>1<br>Time / us

Compound [**1**(**2D**)$_3$]:

Identity confirmation:		*See XRD data (and DEER)*
Sample conditions:	Concentration	0.2 mM
	Tube diameter	3 mm
	Sample volume	0.1-0.2 ml
	Cryoprotectant	Liquid N_2
	Freezing procedure	Flash-freezing
	Deuteration	*None*
	Solvent	Toluene
	Temperature	3 K
Instrumentation:	Spectrometer	Bruker Elexsys E580
	Resonator	MS3 (split-ring)
EPR Parameters:	Pump π-pulse length	10 ns
	Det π/2-pulse length	10 ns
	Det π-pulse length	20 ns
	Pump frequency	9.28 GHz
	Det frequency	9.43 GHz
	Pump-det offset	-150 MHz
	Pump pulse shape	Rectangular
	Det pulse shape	Rectangular
	τ_1 length	300 ns
	τ_2 length	1000 ns
	Shot repetition time	1020 μs
	Time increment	4 ns
	Accumulation time	*See next page*
	Number of averages	*See next page*
Nuclear modulation averaging procedure:	Tau-averaging (τ_1, ^{1}H)	Time step: 8 ns<br>Number of τ_1 to average: 8
Measures to reduce multi-spin effects:		*None as multi-spin effects assumed to be negligible at X-band (low inversion efficiencies)*
Data parameters:	Modulation depth	*See next page*
	SNR (wrt mod depth)	*See next page*
	Zero-time offset	100 ns

τ₂ = 1000 ns	**3690 G**	**3730 G**	**3770 G**	**3810 G**	**3850 G**
Accumulation time / hours	41.5	17	14.5	18.5	56
Number of scans	85	35	30	38	115
Shots per point	50	50	50	50	50
Modulation depth / %	10.7	11.1	9.0	5.2	2.7
SNR wrt MD* / no units	21	53	50	52	14

*calculated using the SnrCalculator program[S5]

Table S5. Normalized raw experimental DEER traces (blue) of [**1**(**2D**)$_3$] for fast and slow sample cooling prior to background correction, overlaid with the corresponding background (dashed red) modelled as a homogeneous (exponential) decay.

**[1(2D)$_3$]**	$\tau_2 = 1000\ ns$; fast cooling	$\tau_2 = 1000\ ns$; slow cooling
Fast cooling: B = 3690 G<br><br>Slow cooling: B = 3830 G	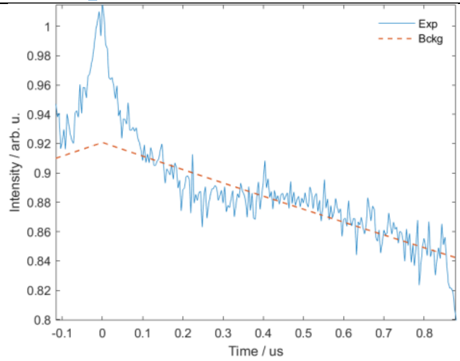	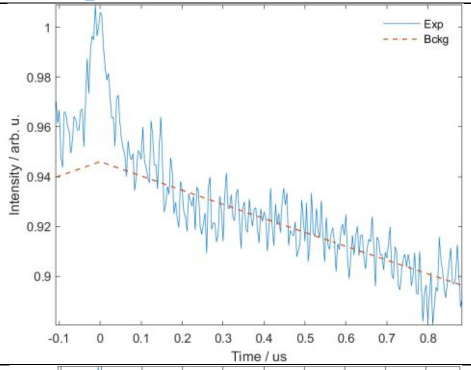
Fast cooling: B = 3730 G<br><br>Slow cooling: B = 3870 G	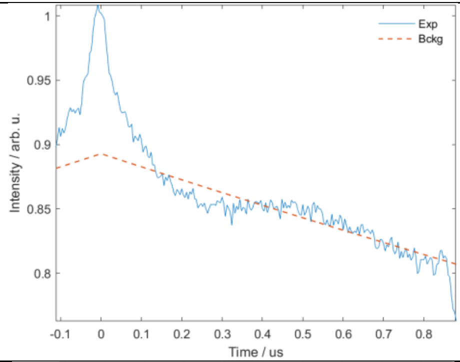	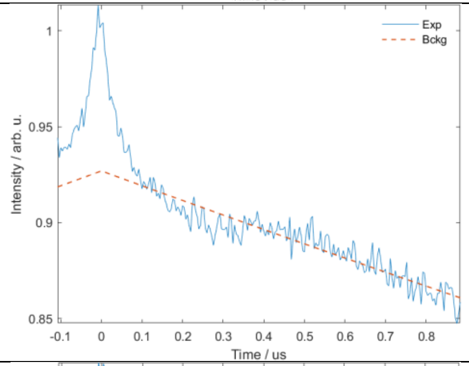
Fast cooling: B = 3770 G<br><br>Slow cooling: B = 3910 G	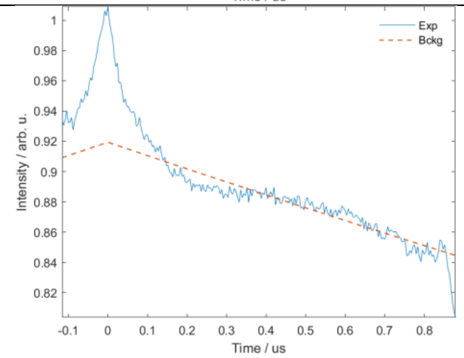	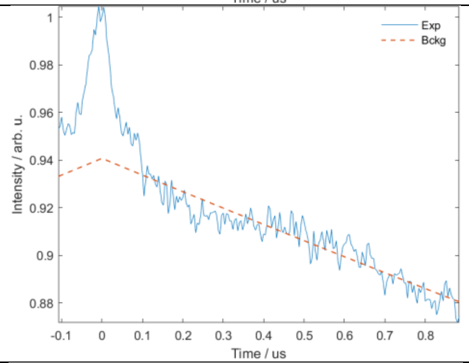
Fast cooling: B = 3810 G<br><br>Slow cooling: B = 3950 G	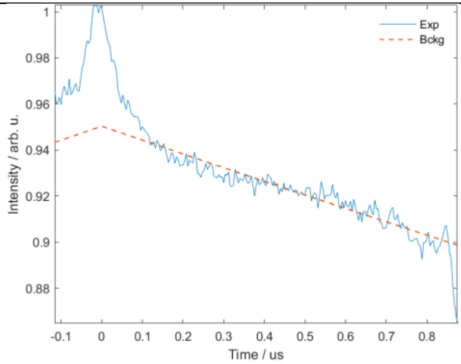	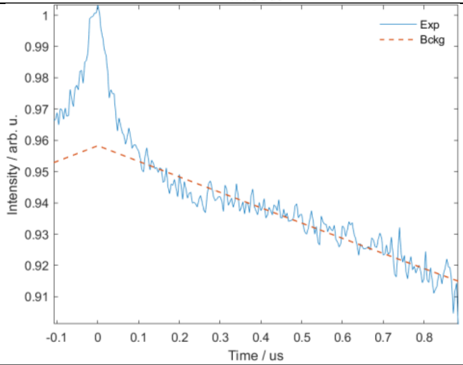

Fast cooling: B = 3850 G<br><br>Slow cooling: B = 3990 G	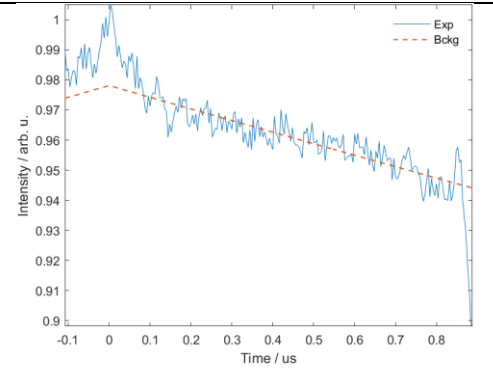	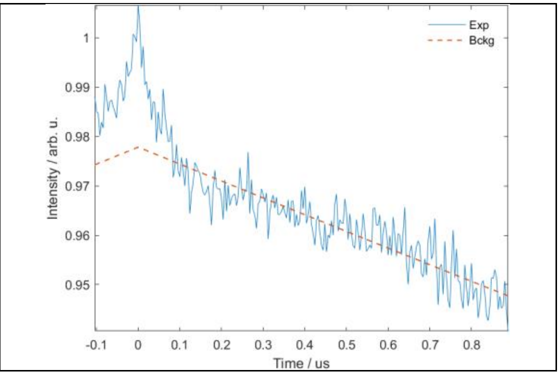

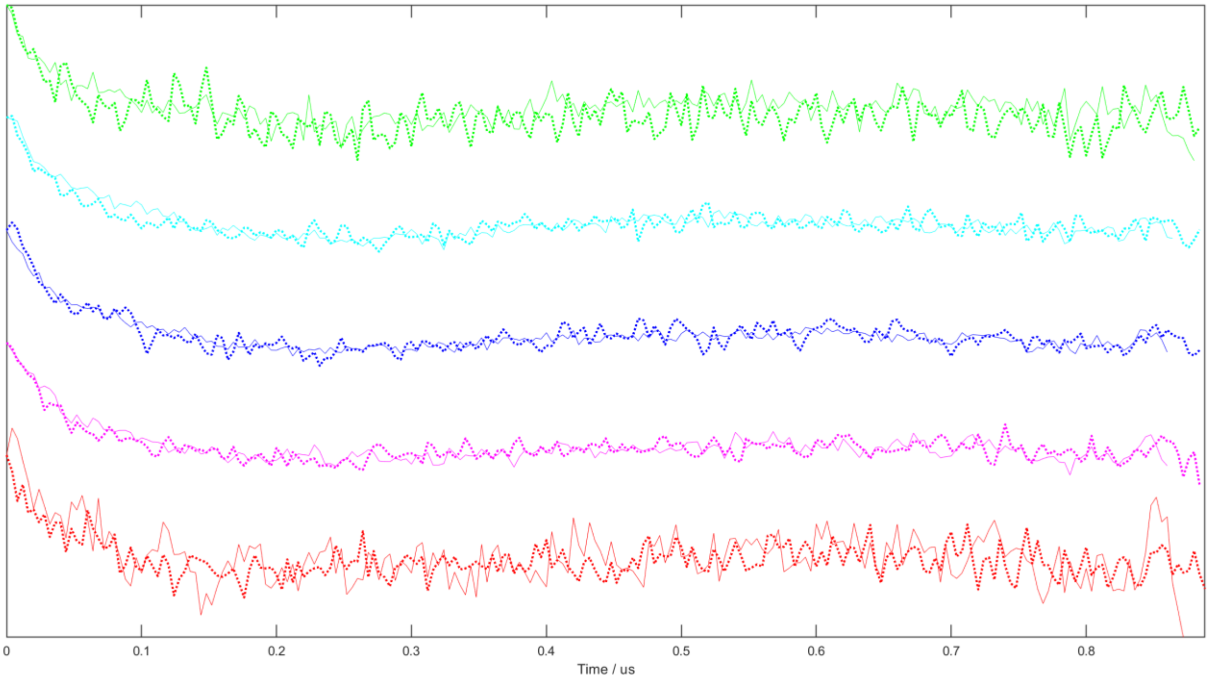

Figure S7. Background-corrected experimental DEER traces of [**1**(**2D**)$_3$] after fast cooling (solid lines) and slow cooling (bold dotted lines) of the sample. The traces are arranged vertically such that the lowest-field traces are at the top and the highest-field traces are at the bottom.

Compound [**1**(**2E**)$_3$]:

Identity confirmation:		*See XRD data (and DEER)*
Sample conditions:	Concentration	0.2 mM
	Tube diameter	3 mm
	Sample volume	0.1-0.2 ml
	Cryoprotectant	Liquid N_2
	Freezing procedure	Flash-freezing
	Deuteration	*None*
	Solvent	Toluene
	Temperature	3 K
Instrumentation:	Spectrometer	Bruker Elexsys E580
	Resonator	MS3 (split-ring)
EPR Parameters:	Pump π-pulse length	10 ns
	Det π/2-pulse length	10 ns
	Det π-pulse length	20 ns
	Pump frequency	9.26 GHz
	Det frequency	9.41 GHz
	Pump-det offset	-150 MHz
	Pump pulse shape	Rectangular
	Det pulse shape	Rectangular
	τ_1 length	120 ns
	τ_2 length	*See next page*
	Shot repetition time	2040 μs
	Time increment	4 ns
	Accumulation time	*See next page*
	Number of averages	*See next page*
Nuclear modulation averaging procedure:	Tau-averaging (τ_1, ^{1}H)	Time step: 8 ns<br>Number of τ_1 to average: 8
Measures to reduce multi-spin effects:		*None as multi-spin effects assumed to be negligible at X-band (low inversion efficiencies)*
Data parameters:	Modulation depth	*See next page*
	SNR (wrt mod depth)	*See next page*
	Zero-time offset	80 ns

τ_2 = 800 ns	**3680 G**	**3720 G**	**3760 G**	**3800 G**	**3840 G**
Accumulation time / hours	10	8.5	8.5	8.5	10
Number of scans	30	25	25	25	30
Shots per point	20	20	20	20	20
Modulation depth / %	19.0	26.7	22.4	13.2	5.1
SNR wrt MD* / no units	68	165	202	134	40

τ_2 = 1200 ns	**3680 G**	**3720 G**	**3760 G**	**3800 G**	**3840 G**
Accumulation time / hours	22	12	12	12	19.5
Number of scans	45	25	25	25	40
Shots per point	20	20	20	20	20

*calculated using the SnrCalculator program[S5]

Table S6. Normalized raw experimental DEER traces (blue) of [**1**(**2E**)$_3$] at two different τ_2 values prior to background correction, overlaid with the corresponding background (dashed red) modelled as a homogeneous (exponential) decay.

**[1(2E)$_3$]**	$\tau_2 = 800\ ns$	$\tau_2 = 1200\ ns$
B = 3680 G	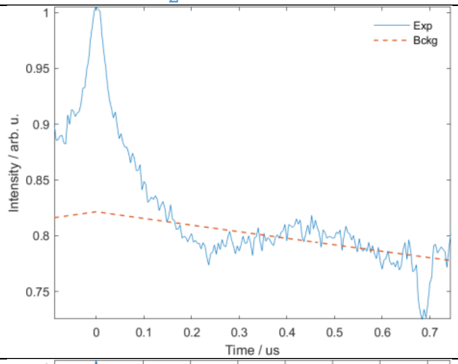	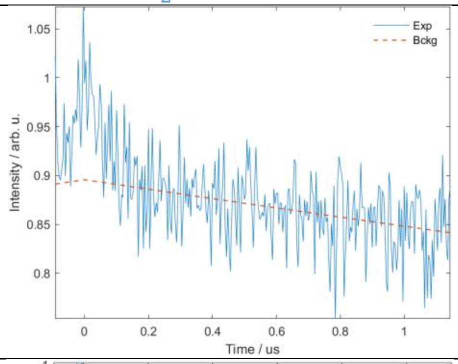
B = 3720 G	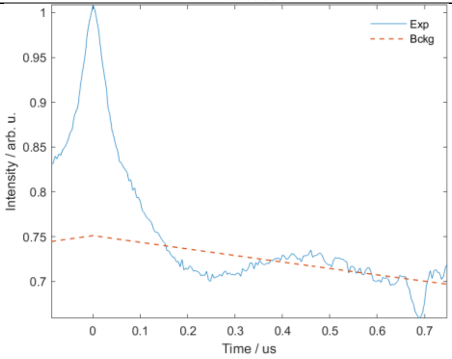	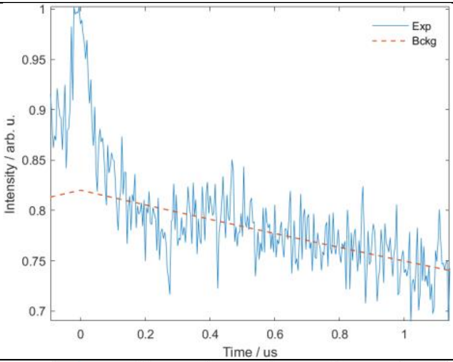
B = 3760 G	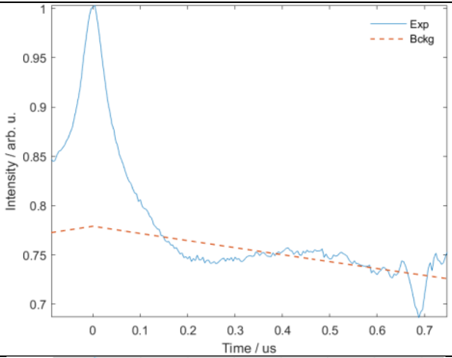	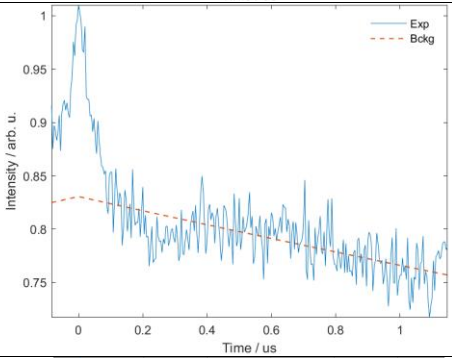
B = 3800 G	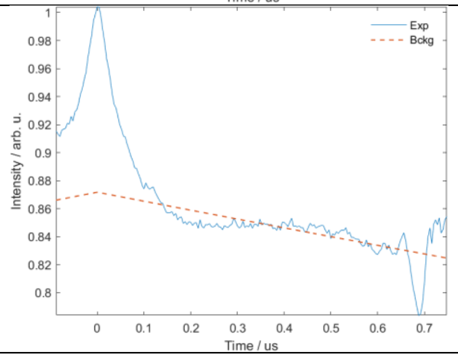	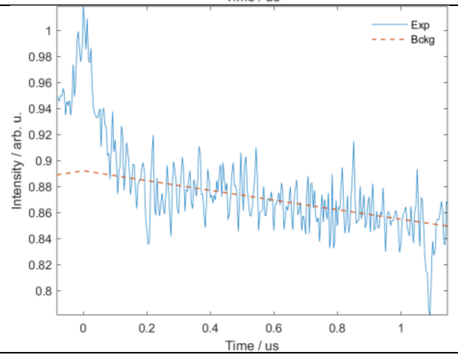

B = 3840 G	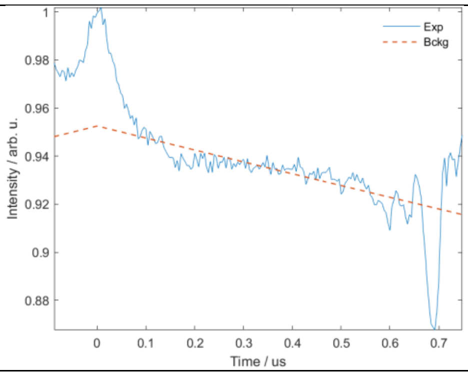Exp<br>Bckg<br>Intensity / arb. u.<br>Time / us<br>1<br>0.98<br>0.96<br>0.94<br>0.92<br>0.9<br>0.88<br>0<br>0.1<br>0.2<br>0.3<br>0.4<br>0.5<br>0.6<br>0.7	

Section 7 – Geometric Flexibility Model:

A model assuming completely free (360°) rotation about each of the three M^{Tri}…N^{Py} coordinate bonds (Figure S8) was employed as the basis for all acceptable conformations simulated by the program. After an initial isotropic generation of 5000 conformations, each conformation was screened and only accepted if all three ring centres in the conformation are in the volume region where the angle of the thread's long axis with respect to the $\{CrNi_2\}$ plane falls within the 60° ± 20° range. At this point, any conformations where the $\{Cr_7Ni\}$ rings were overlapping with each other were also excluded. For each ring, the *z*-axis of the *g*-tensor (g_z) was assigned to the normal of the plane of the ring, in accordance with previous work.[S6,S7] Due to the molecular symmetry present in the rings, the *x*- and *y*- axes were allowed to lie anywhere in the plane of the ring, as long as they were orthogonal to each other.

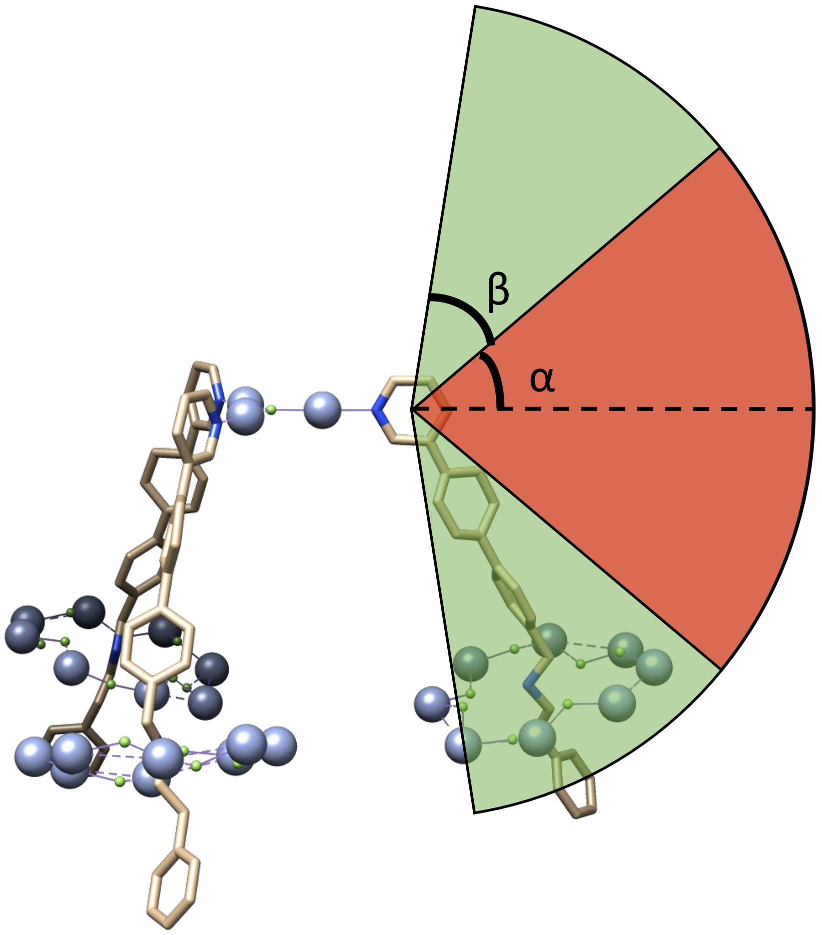

Figure S8. A representation of the model employed in the generation of acceptable conformations (shown here with the crystal structure of [**1**(**2B**)$_3$] for one of the three threads); the green area represents the 3-D volume one of the three ring centres must occupy, with the red region representing an area excluded from this, *i.e.* where no ring centres must be present (α = β = 40°).

In the next step of the fitting process, DEER time traces were simulated for all accepted conformations, and subsequently fitted to the experimental time traces via an iterative least-squares fitting algorithm. In the first fitting cycle, the algorithm determines the conformation whose simulated DEER traces have the smallest sum of the squares of the residuals (*i.e.* 'least squares') from the experimental DEER traces – this is the best-fitting conformation in the first cycle.

The best-fitting conformation for the first cycle is recorded and its simulated DEER traces are subsequently added to the traces of each conformation in the simulation library; these sums of traces are fitted against the experimental traces in the next fitting cycle in the same manner (least-squares fitting) to determine the next best-fitting conformation. Once the next best-fitting conformation is determined, its simulated DEER traces are similarly added to the trace sums of each conformation in the library. This process repeats itself with every cycle; the number of fitting cycles is chosen so that the fit reaches a point whereby adding additional conformations no longer significantly improves the fit.

Since the experimental DEER traces collected generally do not agree well with the simulated DEER traces based on the crystal structures of each compound (see Figure S9), the simulation of a large number of potential conformations and the least-squares fitting of their calculated DEER traces to the experimental data was necessary in order for simulations to match experiment.

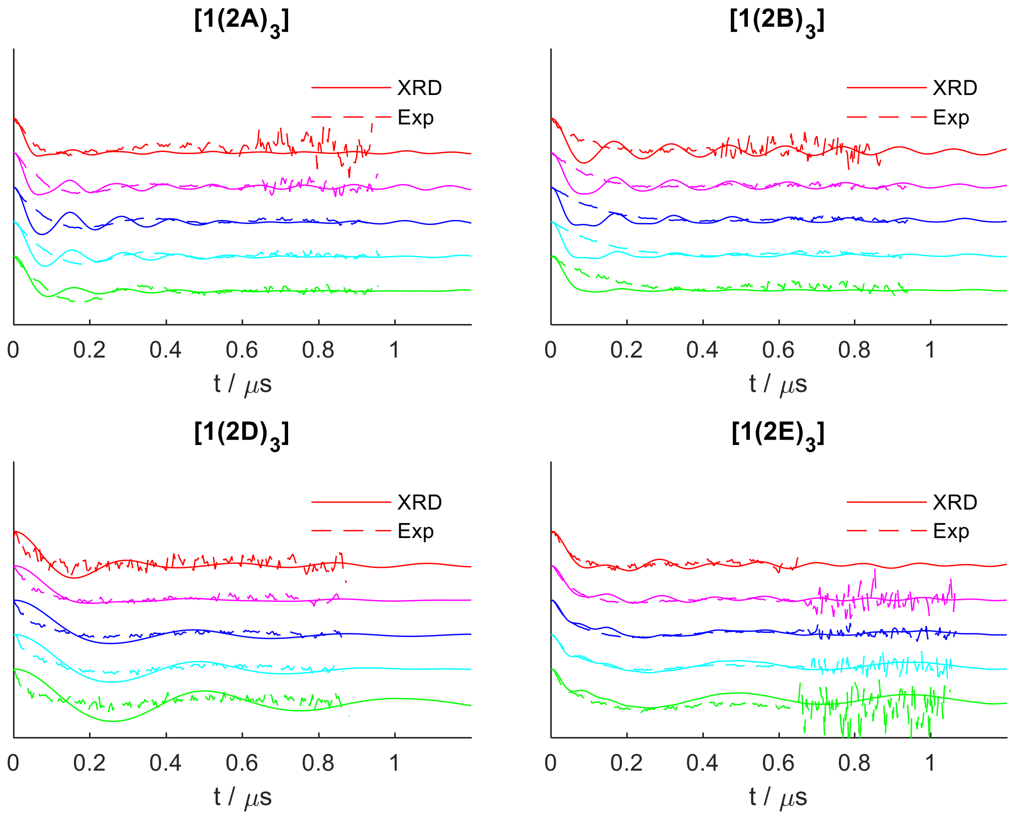

Figure S9. Simulated DEER traces calculated based on the crystal structures of compounds [**1**(**2N**)$_3$] where **N** = {**A**, **B**, **D**, **E**}, overlaid with the experimental DEER traces measured for the compounds at five different field values.

Section 8 – Conformational Analysis Figures:

Compound [**1**(**2B**)$_3$]:

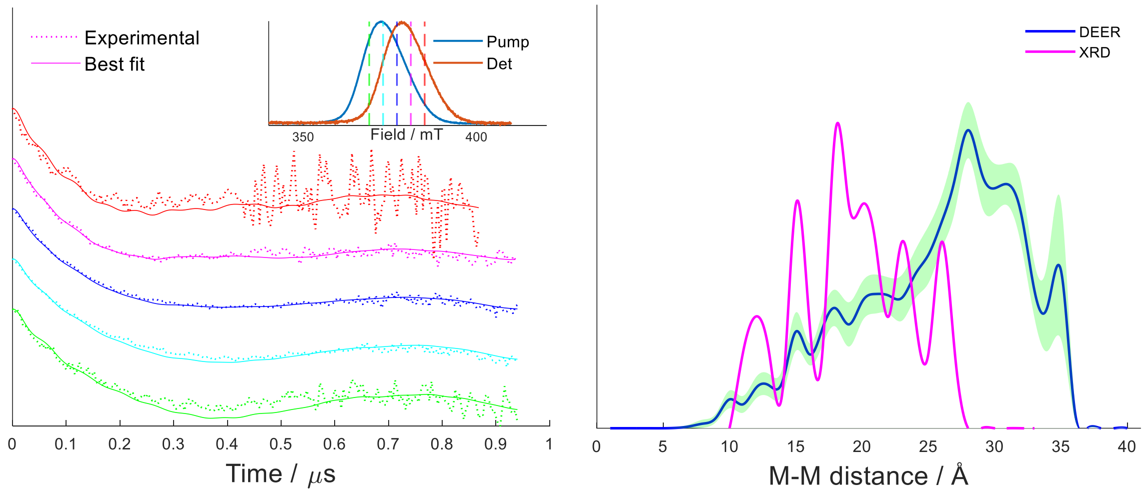

Figure S10a (left). Normalized experimental DEER time traces of [**1**(**2B**)$_3$] overlaid with the simulated conformation fits. Inset: experimental field sweep spectra obtained for [**1**(**2B**)$_3$] at the pump and detection frequencies, with each dashed line indicating the field position of the time trace with the same color. Figure S10b (right). Inter-ring metal-metal distance distributions of [**1**(**2B**)$_3$] (smoothed using cubic-spline interpolation in MATLAB) in solution (blue, sum of best-fitting 50 conformations) and in its crystal structure (magenta, normalized to the maximum of the solution-phase distribution sum); the 95% confidence range for the solution-phase distribution is highlighted in green.

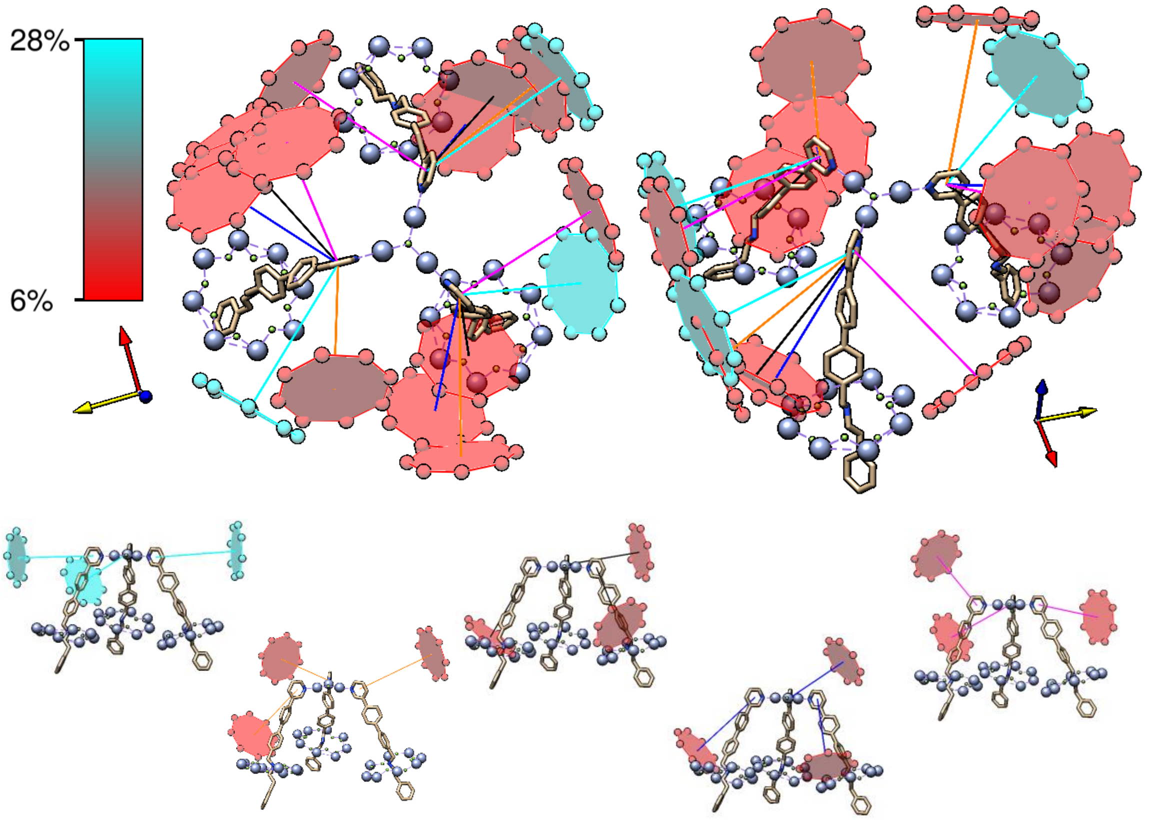

Figure S11. Crystal structure of [**1**(**2B**)$_3$] overlaid with the most dominant (≥6%) solution-phase conformations (shown from two different points of view, as well as separate individual conformations below), with the ring color reflecting the abundance of the conformation in the fit (see color key on the left) and the organic threads of each conformation depicted by lines of the same color; the g-frame of the central {$CrNi_2$} triangle is shown by the axes alongside each structure (x; red, y: yellow, z: blue).

Compound [**1**(**2D**)$_3$]:

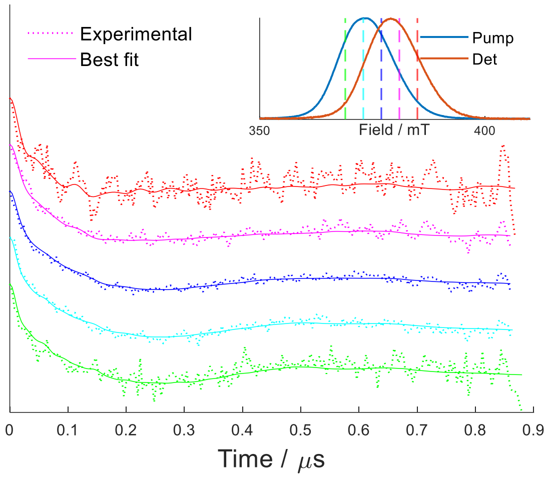

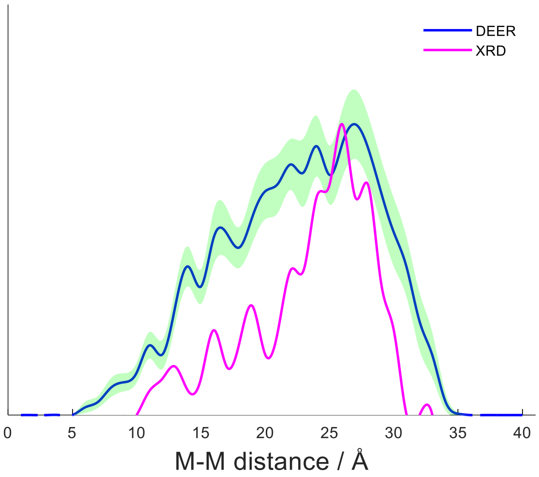

Figure S12a (left). Normalized experimental DEER time traces of [**1**(**2D**)$_3$] overlaid with the simulated conformation fits. Inset: experimental field sweep spectra obtained for [**1**(**2D**)$_3$] at the pump and detection frequencies, with each dashed line indicating the field position of the time trace with the same color. Figure S12b (right). Inter-ring metal-metal distance distributions of [**1**(**2D**)$_3$] (smoothed using cubic-spline interpolation in MATLAB) in solution (blue, sum of best-fitting 50 conformations) and in its crystal structure (magenta, normalized to the maximum of the solution-phase distribution sum); the 95% confidence range for the solution-phase distribution is highlighted in green.

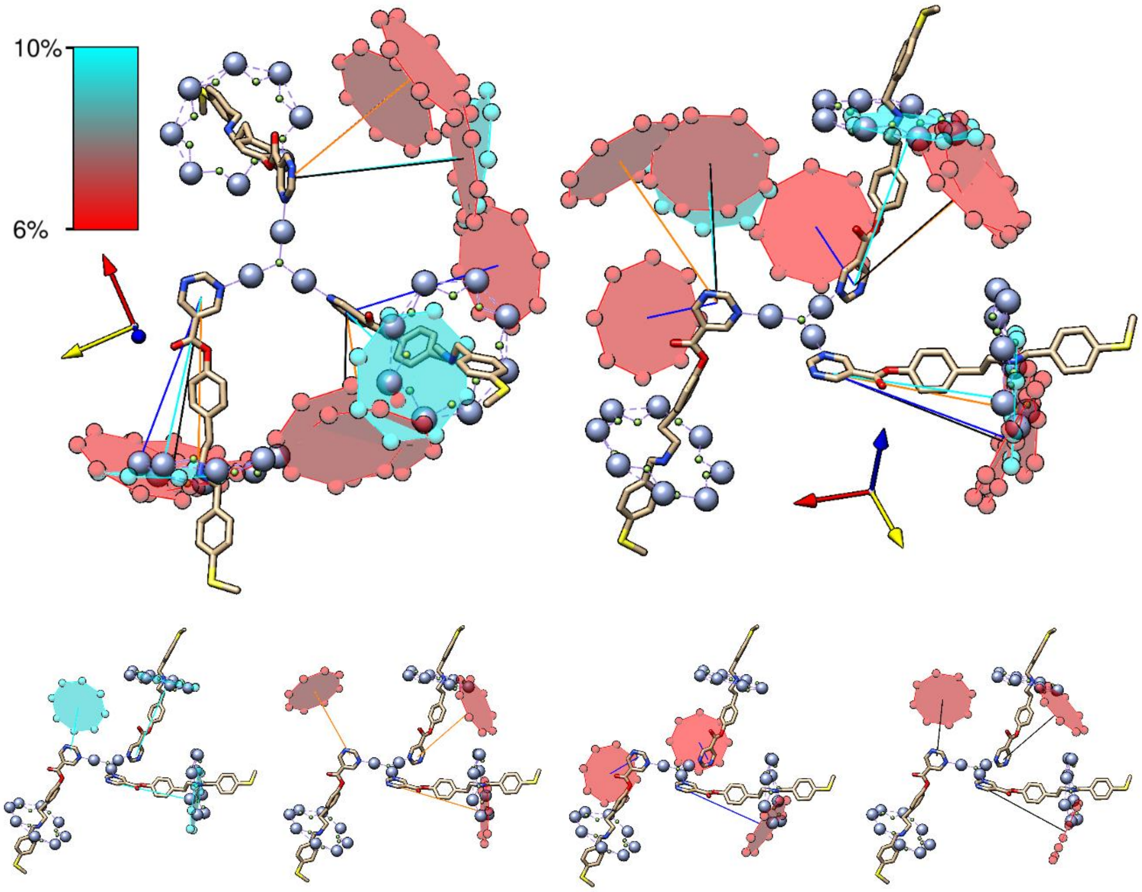

Figure S13. Crystal structure of [**1**(**2D**)$_3$] overlaid with the most dominant (≥6%) solution-phase conformations (shown from two different points of view, as well as separate individual conformations below), with the ring color reflecting the abundance of the conformation in the fit (see color key on the left) and the organic threads of each conformation depicted by lines of the same color; the g-frame of the central {$CrNi_2$} triangle is shown by the axes alongside each structure (x; red, y: yellow, z: blue).

Compound [**1**(**2E**)$_3$]:

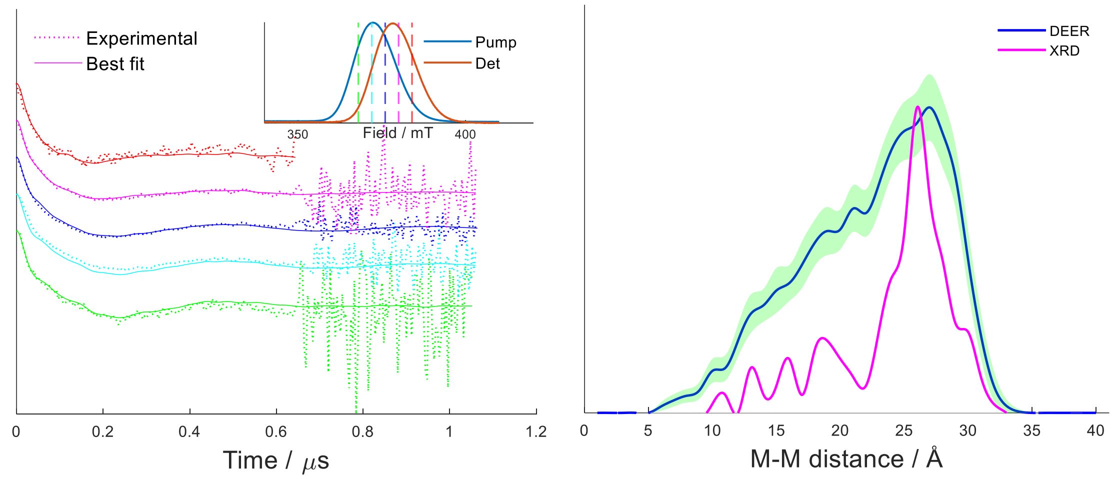

Figure S14a (left). Normalized experimental DEER time traces of [**1**(**2E**)$_3$] overlaid with the simulated conformation fits. Inset: experimental field sweep spectra obtained for [**1**(**2E**)$_3$] at the pump and detection frequencies, with each dashed line indicating the field position of the time trace with the same color. Figure S14b (right). Inter-ring metal-metal distance distributions of [**1**(**2E**)$_3$] (smoothed using cubic-spline interpolation in MATLAB) in solution (blue, sum of best-fitting 50 conformations) and in its crystal structure (magenta, normalized to the maximum of the solution-phase distribution sum); the 95% confidence range for the solution-phase distribution is highlighted in green.

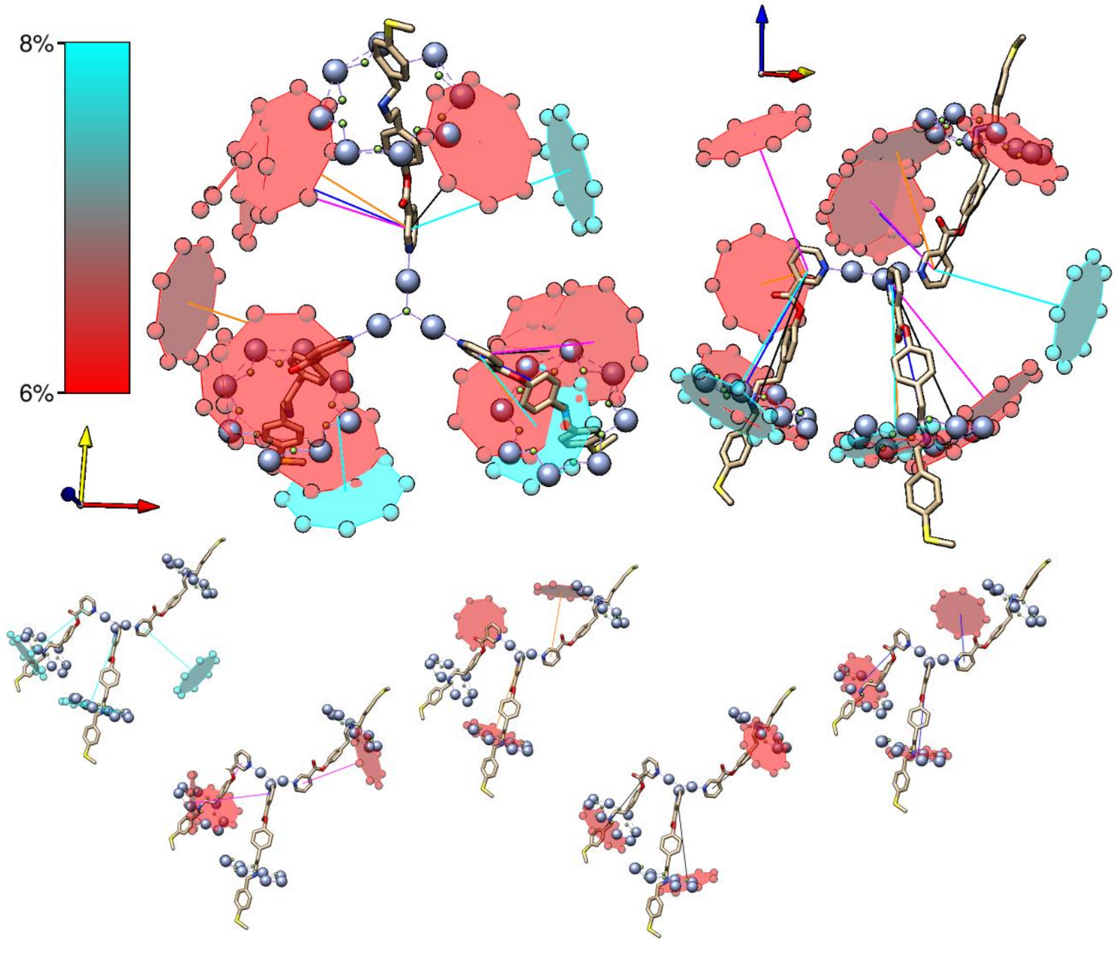

Figure S15. Crystal structure of [**1**(**2E**)$_3$] overlaid with the most dominant (≥6%) solution-phase conformations (shown from two different points of view, as well as separate individual conformations below), with the ring color reflecting the abundance of the conformation in the fit (see color key on the left) and the organic threads of each conformation depicted by lines of the same color; the g-frame of the central {$CrNi_2$} triangle is shown by the axes alongside each structure (x; red, y: yellow, z: blue).

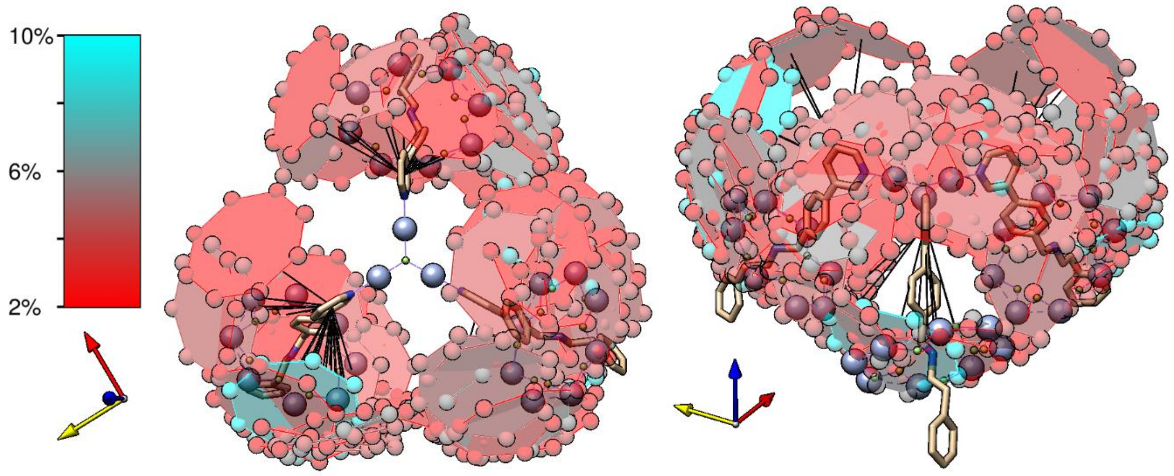

Figure S16. Crystal structure of [**1**(**2A**)$_3$] overlaid with all dominant (≥2%) solution-phase conformations (shown from two different points of view), with the ring color reflecting the abundance of the conformation in the fit (see color key on the left) and the organic threads of each conformation depicted by black lines; the g-frame of the central {$CrNi_2$} triangle is shown by the axes alongside each structure (x; red, y: yellow, z: blue).

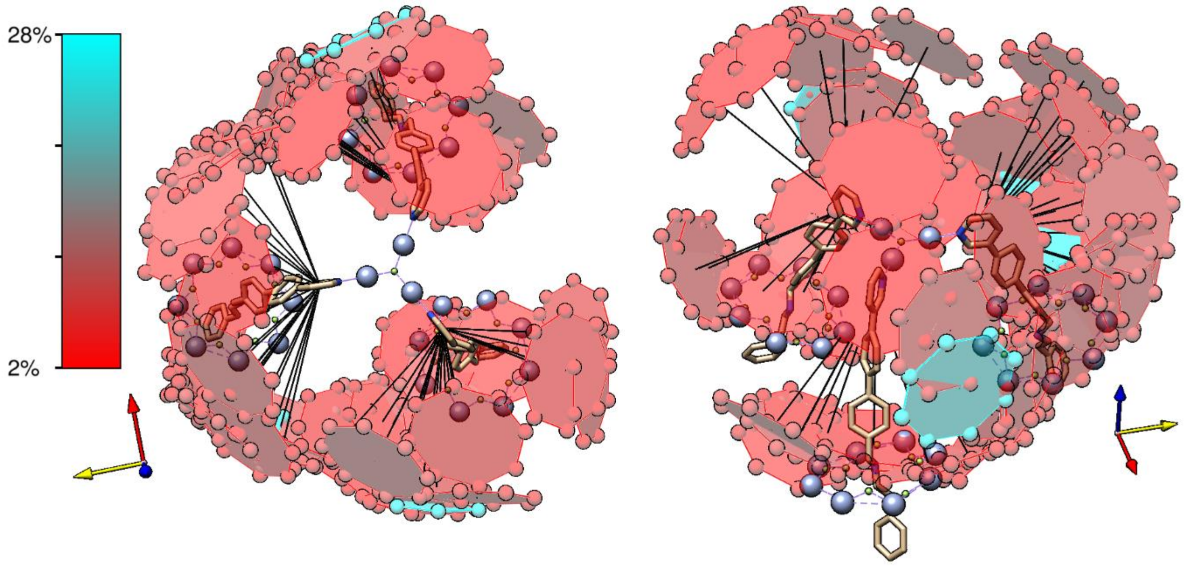

Figure S17. Crystal structure of [**1**(**2B**)$_3$] overlaid with all dominant (≥2%) solution-phase conformations (shown from two different points of view), with the ring color reflecting the abundance of the conformation in the fit (see color key on the left) and the organic threads of each conformation depicted by black lines; the g-frame of the central {$CrNi_2$} triangle is shown by the axes alongside each structure (x; red, y: yellow, z: blue).

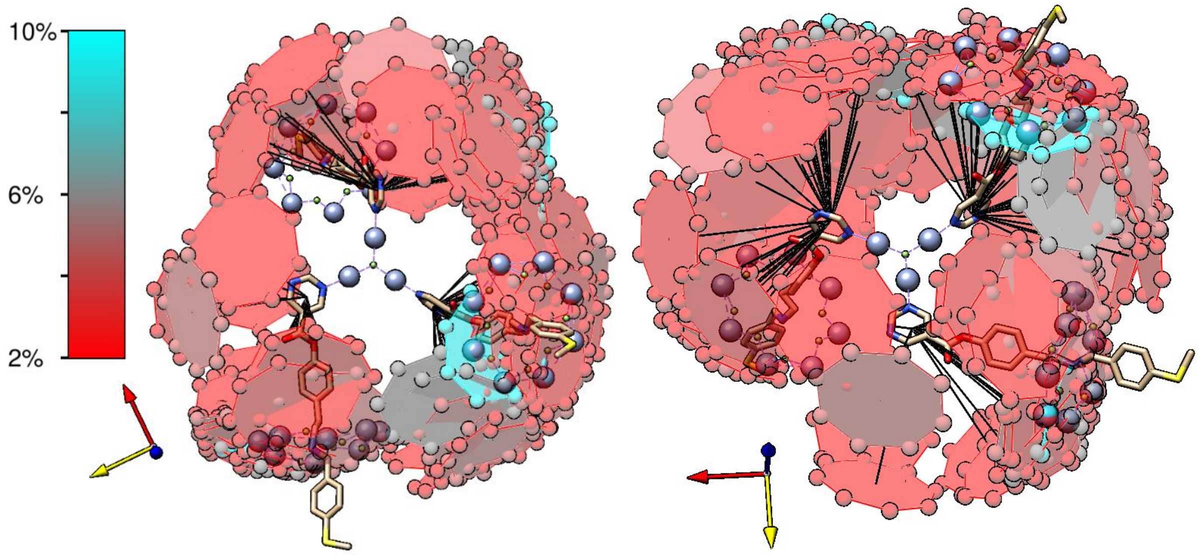

Figure S18. Crystal structure of [**1**(**2D**)$_3$] overlaid with all dominant (≥2%) solution-phase conformations (shown from two different points of view), with the ring color reflecting the abundance of the conformation in the fit (see color key on the left) and the organic threads of each conformation depicted by black lines; the g-frame of the central {$CrNi_2$} triangle is shown by the axes alongside each structure (x; red, y: yellow, z: blue).

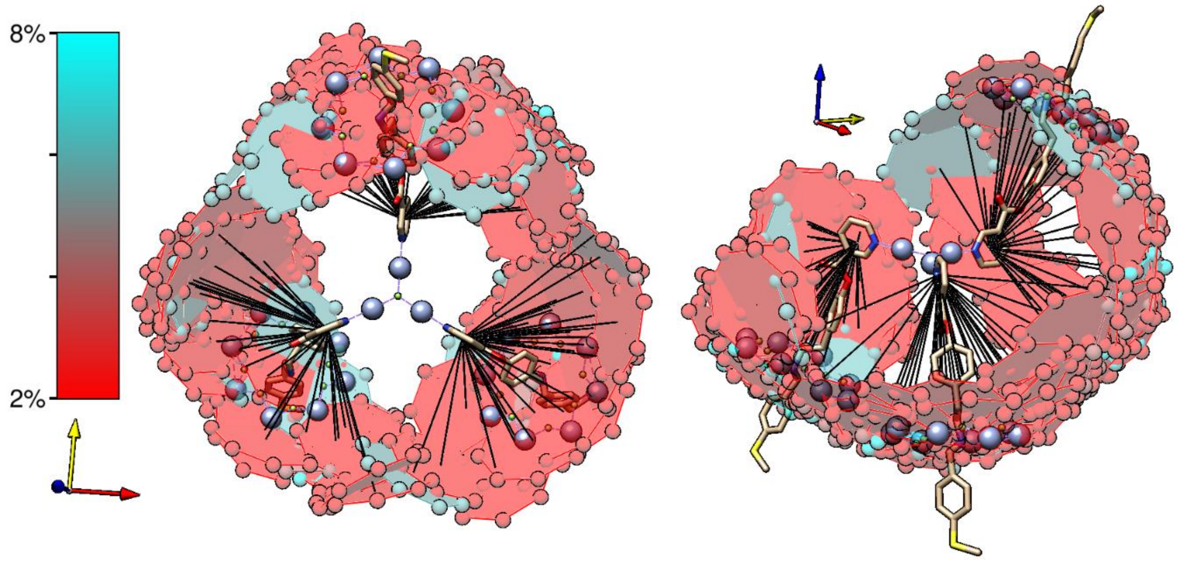

Figure S19. Crystal structure of [**1**(**2E**)$_3$] overlaid with all dominant (≥2%) solution-phase conformations (shown from two different points of view), with the ring color reflecting the abundance of the conformation in the fit (see color key on the left) and the organic threads of each conformation depicted by black lines; the g-frame of the central {$CrNi_2$} triangle is shown by the axes alongside each structure (x; red, y: yellow, z: blue).

Section 9 – Earth Mover's Distance:

The Earth Mover's Distance (EMD) is a statistical parameter that quantifies the dissimilarity between two distributions. As its name suggests, it can be thought of as the minimum work required to transform one arrangement of dirt into another (see Figure S20).

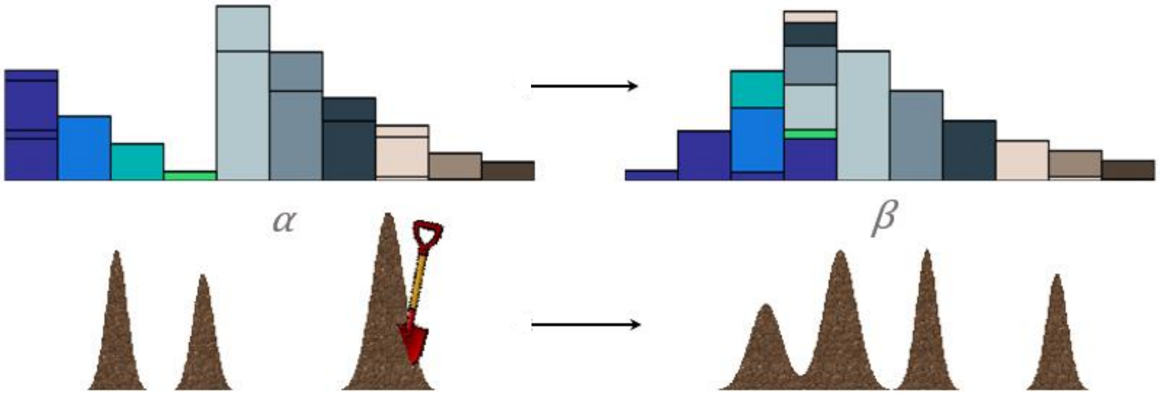

Figure S20. A visualization of the EMD, depicting a rearrangement of one distribution (α) into another (β), similarly to the rearrangement of one set of dirt piles into another set.

This can be formulated as a linear programming problem where the work required to transform one histogram distribution into another is minimized, subject to certain constraints.[S8] For example, if we let $P = \{(p_1, v_1), \ldots, (p_m, v_m)\}$ be the first histogram distribution with m bins – where p_i is the ith bin and v_i is the weight (or frequency) of the ith bin – and $Q = \{(q_1, w_1), \ldots, (q_n, w_n)\}$ be the second histogram distribution with n bins – where q_j is the jth bin and w_j is the weight (or frequency) of the jth bin – then the minimum work required to transform distribution P into distribution Q can be calculated, provided the ground distance matrix $\boldsymbol{D} = [d_{ij}]$ is known, where d_{ij} is the ground distance between bins p_i and q_j.

The objective is to find a flow $\boldsymbol{F} = [f_{ij}]$ – with f_{ij} being the flow between p_i and q_j – that minimises the overall work required for the transformation of P into Q

$$W(P, Q, \boldsymbol{F}) = \sum_{i=1}^{m} \sum_{j=1}^{n} d_{ij} f_{ij}$$

subject to the following constraints:

$$f_{ij} \geq 0 \qquad 1 \leq i \leq m, 1 \leq j \leq n \qquad (1)$$

$$\sum_{j=1}^{n} f_{ij} \leq v_i \qquad 1 \leq i \leq m \tag{2}$$

$$\sum_{i=1}^{m} f_{ij} \leq w_j \qquad 1 \leq j \leq n \tag{3}$$

$$\sum_{i=1}^{m} \sum_{j=1}^{n} f_{ij} = \min(\sum_{i=1}^{m} v_i , \sum_{j=1}^{n} w_j) \tag{4}$$

As distribution P is transformed into distribution Q and not vice versa, constraint (1) ensures that only the constituent elements of P are reallocated into distribution Q rather than the other way round. Constraints (2) and (3) are mutually related: constraint (2) ensures the amount reallocated into distribution Q from bin i (of distribution P) does not exceed the weight of bin i (v_i), while constraint (3) ensures the amount from distribution P reallocated into bin j (of distribution Q) does not exceed the weight of bin j (w_j). Finally, constraint (4) forces the solution to reallocate the maximum possible amount of the constituent elements of distribution P into distribution Q – this is known as the total flow. Once the solution is found (giving the optimal flow $\boldsymbol{F}$), the EMD is defined as the work done transforming P into Q, normalized by the total flow:

$$EMD(P,Q) = \frac{\sum_{i=1}^{m} \sum_{j=1}^{n} d_{ij} f_{ij}}{\sum_{i=1}^{m} \sum_{j=1}^{n} f_{ij}}$$

All EMD calculations were performed using a custom MATLAB-based linear programming algorithm written by Ulas Yilmaz.[S9] The raw EMD values were scaled by factors equal to the quotients of the mean triangle-ring distance for [**1**(**2A**)$_3$] and the analogous mean distance for each of the four compounds (see main paper), given in Table S7.

Table S7. Mean triangle-ring distances for each compound, measured from the center of the ring to the central fluoride in the triangle (rounded to 3 d. p.), along with scaling factors that the EMD value for each compound was multiplied by to remove thread length dependence (rounded to 4 d. p.).

Compound	Mean triangle-ring distance / Å	Scaling factor
[**1**(**2A**)$_3$]	12.090	1.0000
[**1**(**2B**)$_3$]	15.202	0.7953
[**1**(**2D**)$_3$]	14.116	0.8565
[**1**(**2E**)$_3$]	14.351	0.8425

Section 10 – Angular Earth Mover's Distance:

As described in the main paper, the Earth Mover's Distance (EMD) can also be calculated by considering the rotational/angular differences between conformations rather than purely the inter-conformational distances/separations. However, an intrinsic limitation in effectively reducing a two-dimensional problem to a single dimension can lead to incorrect EMD values: if two vectors exist in the *xy*-plane where the angles between the vectors and the *x*-axis have opposite signs to each other due to the convention used for defining the angles (Figure S21a), the calculated angular EMD value can be considerably greater than the true value. This can also be visualized in the context of rotating one vector to align with the other, where rotation can only occur in one direction and so the rotation needed to align one vector with the other is greater than it would be if the vector was rotated in the opposite direction.

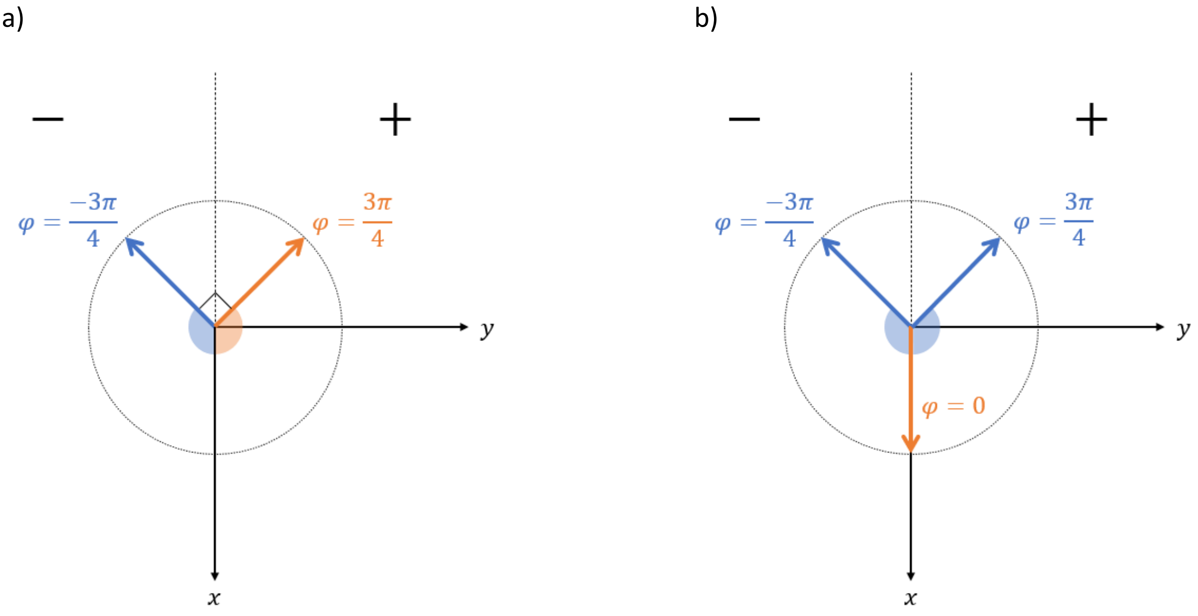

Figure S21a (left). A representation of the error that can arise in the angular EMD calculation between vector 1 (orange arrow) and vector 2 (blue arrow) – as the sign of angle ϕ depends on which side of the *x*-axis the vector is located on, the angular difference between the two is calculated to be $3\pi/2$, even though the minimum angular difference is in fact $\pi/2$. Figure S21b (right). A representation of the special case where this error is absent – if the angle ϕ of vector 1 (orange arrow) is set to 0, then the minimum angular difference between vector 1 and any other vector (blue arrows) is always calculated correctly by the EMD algorithm, regardless of the sign of the angle ϕ of the other vector.

This error is removed if the coordinate frame is defined such that the angle between one vector and the *x*-axis is equal to zero (Figure S21b), as then the angular difference between the two vectors will simply be equal to the absolute value of the angle between the other vector and the *x*-axis, regardless of the sign of this angle. Nevertheless, while we have calculated angular EMD values between the crystal structures and the solution-phase conformations where the ϕ angles of the crystal structures are equal to zero in all cases, calculating angular EMD values for fit validation would involve the comparison of multiple sets of 50 conformations (rather than comparing one set of 50 conformations to one crystal structure). In the latter case, it is unclear how this error could be removed (not all 50 conformations in a set can have their angles ϕ defined as 0), hence we have refrained from calculating angular EMD values for testing of the fits for robustness. Future work could consider other representations of these angular orientations that are not subject to this choice of frame – such as utilizing unit quaternions to describe the rotations (which map 3D rotations with their inherent periodicity into a continuous four-dimensional space), followed by computing the geodesic distance between quaternion orientations as a metric of angular deviation.[S10]

Section 11 – References:

(S1) A. Fernandez, E. M. Pineda, J. Ferrando-Soria, E. J. L. McInnes, G. A. Timco, R. E. P. Winpenny. A hybrid organic-inorganic molecular daisy chain. *Chem. Commun.* **2015**, *51*, 11126-11129.

(S2) T. Y. Kim, L. Digal, M. G. Gardiner, N. T. Lucas, J. D. Crowley, Octahedral $[Pd_6L_8]^{12+}$ Metallosupramolecular Cages: Synthesis, Structures and Guest-Encapsulation Studies. *Chem. Eur. J.* **2017**, *23*, 15089 – 15097.

(S3) S. J. Lockyer, S. Nawaz, A. Brookfield, A. J. Fielding, I. J. Vitorica-Yrezabal, G. A. Timco, N. A. Burton, A. M. Bowen, R. E. P. Winpenny, E. J. L. McInnes, Conformational Flexibility of Hybrid [3]- and [4]-Rotaxanes. *J. Am. Chem. Soc.* **2020**, *142*, 37, 15941–15949.

(S4) S. Stoll, A. Schweiger, Easyspin, a comprehensive software package for spectral simulation and analysis in EPR. *J. Magn. Reson*. **2006**, *178*, 42-55.

(S5) D. Abdullin, P. Brehm, N. Fleck, S. Spicher, S. Grimme, O. Schiemann, Pulsed EPR Dipolar Spectroscopy on Spin Pairs with one Highly Anisotropic Spin Center: The Low-Spin Fe^{III} Case. *Chem. Eur. J.* **2019**, *25*, 14388-14398.

(S6) S. Piligkos, H. Weihe, E. Bill, F. Neese, H. El Mkami, G. M. Smith, D. Collison, G. Rajaraman, G. A. Timco, R. E. P. Winpenny, E. J. L. McInnes, EPR Spectroscopy of a Family of $Cr^{III}{}_7M^{II}$ (M = Cd, Zn, Mn, Ni) "Wheels": Studies of Isostructural Compounds with Different Spin Ground States. *Chem. Eur. J.* **2009**, *15*, 13, 3152-3167.

(S7) A. Ardavan, A. M. Bowen, A. Fernandez, A. J. Fielding, D. Kaminski, F. Moro, C. A. Muryn, M. D. Wise, A. Ruggi, E. J. L. McInnes, K. Severin, G. A. Timco, C. R. Timmel, F. Tuna, G. F. S. Whitehead, R. E. P. Winpenny, Engineering coherent interactions in molecular nanomagnet dimers. *npj Quantum Information* **2015**, *1*, 15012.

(S8) Y. Rubner, C. Tomasi, L. J. Guibas, The Earth Mover's Distance as a Metric for Image Retrieval. *International Journal of Computer Vision* **2000**, *40*, 99-121.

(S9) Ulas Yilmaz (**2023**). The Earth Mover's Distance (https://www.mathworks.com/matlabcentral/fileexchange/22962-the-earth-mover-s-distance), MATLAB Central File Exchange. Retrieved July 24, 2023

(S10) D. Q. Huynh, Metrics for 3D Rotations: Comparison and Analysis. *J. Math. Imaging Vis.* **2009**, *35*, 155-164.
